# A missing continental impact melt sheet signal in the early Earth detrital zircon record

**DOI:** 10.1038/s41467-026-76529-w

**Published:** 2026-08-11

**Authors:** Ian Szumila, Dustin Trail, Miki Nakajima, Veanessya Vazquez-Lopez, Rasmus Haugaard, Taus Jørgensen, Michael R. Ackerson, Steven B. Shirey, Nicolas B. Litza, Scott Hull, James Darling, Shweta Narkar

**Affiliations:** 1 Carnegie Institution for Science, Washington, DC, USA; 2 Department of Earth and Environmental Sciences, University of Rochester, Rochester, NY USA; 3 Laurentian University, Sudbury, ON Canada; 4 Smithsonian Institution, Washington, DC, USA; 5 Institute of the Earth & Environment, University of Portsmouth, Portsmouth, UK; 6 Rensselaer Astrobiology Research and Education Center, Rensselaer Polytechnic Institute, Troy, NY USA

**Keywords:** Planetary science, Geochemistry, Geochemistry

## Abstract

Impact-derived melting has been proposed as a significant process in the formation of Earth’s early crust which could mean that impact melts are a source for Earth’s oldest minerals including ancient zircons. However, the source lithology of many Archean and Hadean zircons is unclear because many grains are detrital. Therefore, in this work, we develop a machine learning model trained on the U/Yb, Th/U, Yb, and Gd/Yb values of zircons from the 1.85 Ga Sudbury impact melt sheet and more typical igneous processes, so that it could distinguish between them. Here, we show that ancient zircons from three >3.2Ga localities did not crystallize from impact melt sheets. Our results support the hypothesis that early earth zircons are not sourced directly from impact melt sheets, especially one produced by impacting continental crust and indicate no evidence for a late heavy bombardment implying a more habitable Hadean.

## Abstract (web summary)

Using a combination of machine learning and a large dataset of impact-melt zircons from Sudbury, Szumila et al. find no evidence that Hadean-Archean zircons formed within large impact melt sheets. This finding suggests a more habitable Hadean Earth and a lower impactor flux than previously proposed.

## Introduction

Impacted terrains undergo intense pressure–temperature (P–T) metamorphism, yet on planets with active tectonics and hydrologic cycles, like Earth, even the largest impact melt sheets, such as Vredefort with a crater diameter of ~250 km^1^, can be almost entirely erased from the geologic record over time^2^. Evidence from lunar cratering suggests impactor flux was significantly higher in the first 600 Myr of solar system history^3–6^, indicating that impacts could have contributed to forming Earth’s early crust^7^. Still, direct evidence for the role of impacts in shaping the Earth’s surface during the Hadean remains scarce, due to the limited availability of Hadean samples, which are almost exclusively restricted to detrital zircons. Nevertheless, modeling studies have inferred that impact melt sheets were an important component of Earth’s early crust^8–10^ and the origin of life^11^.

**Fig. 1:**
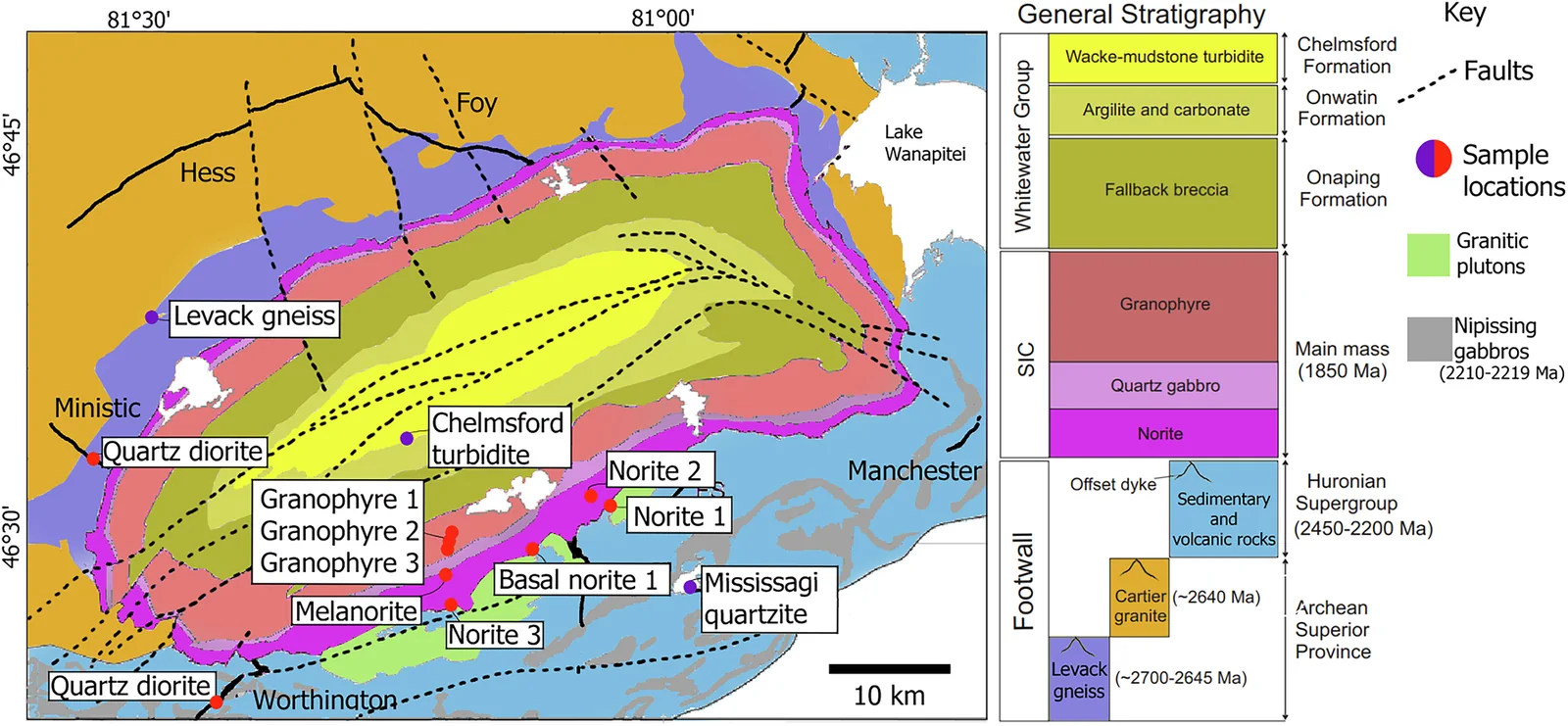
Sudbury impact structure map with stratigraphy. Red circles indicate melt sheet samples yielding zircon analyses used for the SVM. Purple circles denote sampled footwall and infilling lithologies. The impact target rocks include Proterozoic supracrustal units (Huronian Supergroup) and a dominant crystalline basement of Archean Superior Province age (~2700–2640 Ma). The latter, particularly the tonalitic to granodioritic Levack gneiss and Cartier granite, provides a robust geochemical and petrologic analog for the TTG-rich crust characteristic of the Hadean and Eoarchean Earth. This map is adapted and colorized from ref. ^14^ with permission from Elsevier, which is modified from ref. ^63^.

Given the absence of any known Hadean impact melt sheets, rocks, and minerals, younger, well-preserved examples must be used to understand early Earth impact processes. One such example is the 1.85 Ga Sudbury impact structure^12^ (Fig. 1), which has an expansive melt sheet that differentiated into multiple lithologies^13^, and is located in Ontario, Canada. The Sudbury melt sheet is also the largest known preserved impact melt sheet on Earth^14^.

Despite a limited rock record for the early Earth, analyzing the chemistry of Hadean (>4.031 Ga) zircon^8,15^, the oldest known terrestrial minerals, has been informative about Hadean magmatic processes^16^ and whether liquid water was present^15^, which is important for habitability. Trace element concentrations in zircons, governed by crystal-melt partition coefficients, help to constrain parent melt chemistry^17–20^. Zircon Ti concentration has been related to zircon crystallization temperature^21^. For instance, trace element diagrams relying on U vs. Yb, U/Yb vs. Hf, and U/Yb vs. Y can be used to infer whether zircon grains are from oceanic crust or continental crust^22,23^, demonstrating that zircon geochemistry can answer some provenance questions. When these same diagrams were applied to zircons from Vredefort, Sudbury norites, Manicouagan, and Morokweng impact melt sheets, it was found^24^ that melt sheet-derived zircons had indistinguishable REE patterns from other igneous zircons.

However, considerable disagreement persists regarding the geochemical relationship between Sudbury and Hadean zircons, and specifically what these comparisons reveal about crust formation on the early Earth. One study^25^, which measured Ti-in-zircon from Sudbury zircons, found zircon crystallization temperatures differed between Sudbury impact melt sheet zircons and Hadean zircons, and concluded that impact melts were not a Hadean zircon primary source. In contrast, a different study characterized populations of different mineral inclusions in Sudbury zircons, and inferred that Hadean zircons were derived from impact melt^26^. Another analysis, which sampled Sudbury zircon from norites (*n* = 61), quartz gabbro (*n* = 11), and granophyre (*n* = 73) and analyzed Ti in zircon via ion microprobe, found similar melting crystallization temperatures with Hadean zircons, and argued that impact melts are a likely source for Hadean zircons^27^.

To help resolve these conflicting interpretations, we evaluate the trace element signature of impact melt sheet zircons against a global record of endogenous magmatism. Specifically, we compare the trace element chemistry of impact melt sheet zircons with zircons from (I)gneous-, (S)edimentary-, and (A)norogenic- granitoids, which capture the vast majority of endogenous processes for magmatic zircon on Earth^28^. Differences between I- and S-type granites were originally recognized in the Lachlan fold belt^29^, and current machine-learning methods can successfully use trace element measurements to determine whether a zircon originated from an I-, S-, or A-type granite^28^ with accuracies greater than 85%. Generally, S-types form at continent-continent collision zones. Meanwhile, I-type rocks are from continent and island arcs, and A-types are produced from melting within plates^28^. If Hadean zircons are endogenous, the most similar melting process is probably that responsible for I-, S-, and A- granites.

**Fig. 2:**
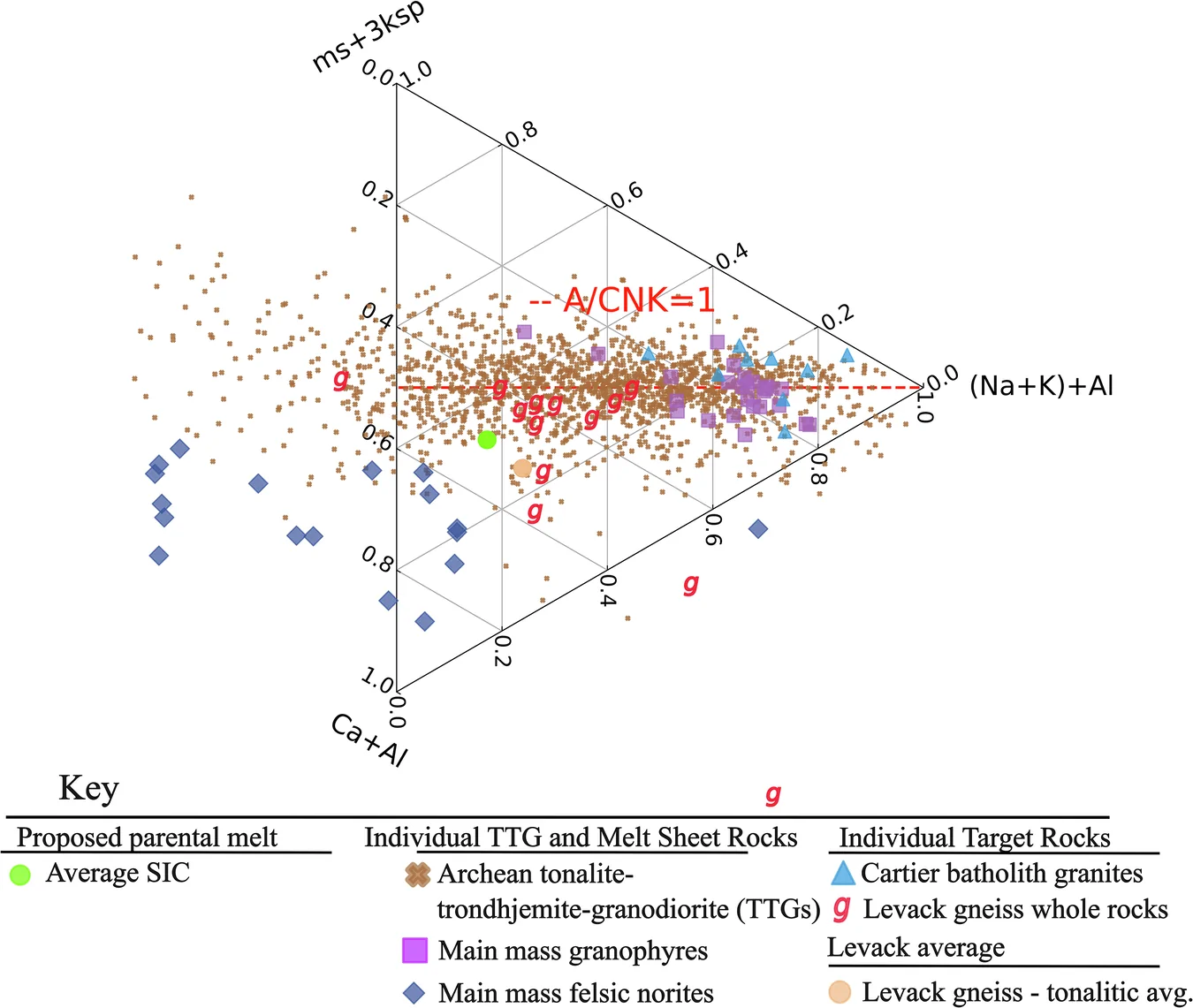
Sudbury melt sheet whole rock compositions compared with potential target rock and early Earth rock geochemistry. All whole rock compositions have been projected from biotite onto a portion of the [Ca+Al - Na+K+Al – 3Al + 2(Na+K)] plane after ref. ^64^. This projection helps to emphasize differences in the compositional sources for rock types while minimizing the input of silica, FeO, and MgO, thus minimizing the effects of differentiation^64^. The compositions of tonalite–trondhjemite–granodiorite (TTG) rocks as reported by Moyen^30^ are also shown. The average Sudbury Igneous Complex (SIC) has been proposed as a parental melt for the Sudbury melt sheet^35,65^. The granophyres from the Sudbury melt sheet plot in a similar region to the Cartier batholith target rock. The average of the Levack gneiss target rock composition plots is close to Average SIC. Many of the Levack gneiss and Cartier batholith rocks can be seen to plot in the same region as the TTG compilation from ref. ^30^. The Levack gneiss tonalitic average shown was reported by Meldrum et al.^31^, while individual Levack gneiss whole rock measurements are from refs. ^66,67^. An extended version of this graph is available as Supplementary Fig. 4, which shows some of the other rock types (e.g., mafic norite) which plot outside the range shown on this graph. ms muscovite, ksp K-feldspar, A/CNK aluminous saturation index.

To explore the suitability of the Sudbury target rocks as analogs for early Earth crust, we compared the whole-rock compositions of the dominant footwall lithologies, such as the Levack gneiss complex and Cartier batholith, with the whole-rock geochemistry of a global compilation of Archean tonalite–trondhjemite–granodiorites (TTGs) reported by Moyen^30^. The petrology of target rocks at Sudbury, a proposed Sudbury parental melt, norite and granophyre rocks from the Sudbury impact melt sheet, and other relevant rocks are compared in Fig. 2. The Cartier batholith, which is composed primarily of granitic rocks rich in potassium^31^, and the Levack gneiss rocks broadly plot in the same compositional space as TTGs (Fig. 2). Given that much of the Levack complex is tonalitic gneiss^31^, this helps to justify our use of the Sudbury impact as an analog to impacts on the early Earth, since TTG material may have been a major component of the continental crust at the time^32^. There has also been proposed evidence for granitic rocks on the Hadean Earth^15,33^, additionally justifying our use of Sudbury as an analogy to a Hadean impact. The SIC granophyre rocks plot near the same compositional region as granites from the Cartier batholith. Norites at Sudbury are often divided into a mafic norite and a unit called “felsic” norite, which contains only about 20% orthopyroxene^34^. Almost all these nodes plot outside the main joins of the graph. In addition, most of the Sudbury mafic norites and the quartz-rich norite (QRN) composition, also proposed as parental melt^35,36^, plot outside the range shown here, so their location can be found in the extended version of this graph found in the supplemental. While the Sudbury granophyre rocks plot in the same region as many of the TTG rocks, the trend for Sudbury norite rocks differs from that of TTGs, indicating divergent compositional pathways between endogenous processes and melt sheets.

In this work, we collect samples and analyze the trace element contents of zircons from the Sudbury impact melt sheet and develop a machine-learning model that predicts whether a zircon crystallized in an impact melt sheet or in an endogenously produced melt. One hurdle was that a large curated dataset of impact melt zircon geochemistry did not exist, so we supplied *n* = 304 additional LA-ICP-MS impact melt zircon analyses. We compile these analyses (Supplementary Data 1), which along with zircon analyses in literature data from endogenous sources, were used to train a support vector machine (SVM). We apply the SVM to classify zircons from three different >3.2 Ga localities, the Jack Hills, the Acasta gneiss, and the Barberton Greenstone Belt, as either derived from an impact melt sheet or from an endogenous origin (i.e., crystallization from typical terrestrial magmatism). Our results support the hypothesis that early Earth zircons (>3.2 Ga) were not derived from impact melt sheets.

## Results

### Zircon trace element contents and geochemical differences

**Fig. 3:**
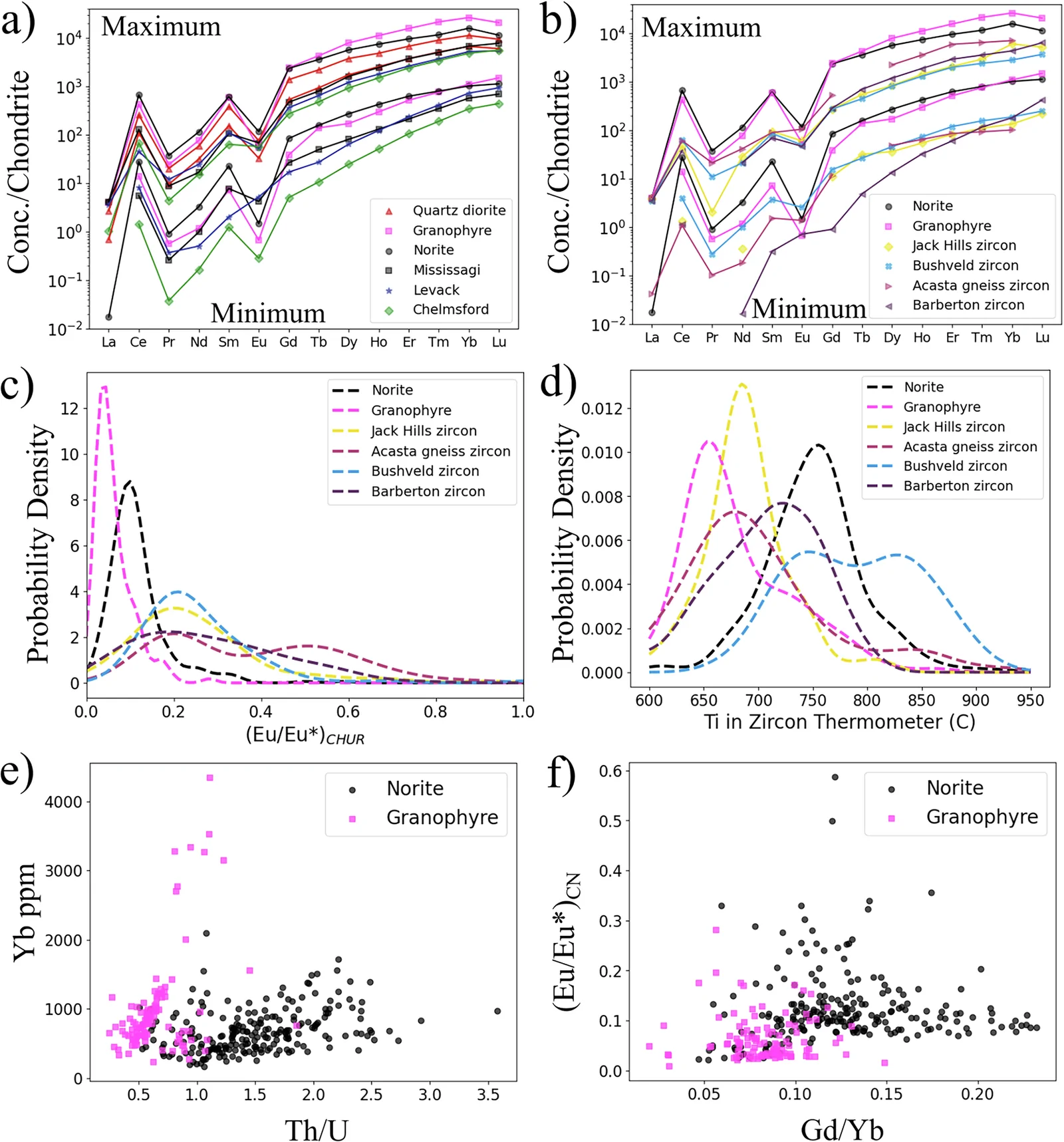
Trace element geochemistry analysis panels for zircons from the Sudbury impact melt sheet and related or early Earth locales. **a** Minimum and maximum REE concentrations in zircons from rocks part of or near the Sudbury impact structure. **b** Plotting the range of possible REE concentrations for Sudbury melt sheet zircons with the ranges for zircons from other localities shows that these zircons have a higher max concentration for most elements, but that minimum concentrations are still within the range of the other localities compared. **a**, **b** Ho and Tm were not analyzed, so concentrations are interpolated. Additionally, to aid visualization on the log scale plots, when conc./chondrite is below 0.01 for an element, it is displayed as 0.01 (10^−2^). No max or minimum (e.g., Barberton La, Ce, Pr) indicates no analyses. **c** The Sudbury norite and granophyre zircons yield the largest negative europium anomalies, i.e., (Eu/Eu*)_CN_ calculated as Eu_CN_/√(Sm_CN_ x Gd_CN_), when compared with Bushveld and early earth zircons with all are plotted as probability density functions (PDFs). **d** The Ti-in-zircon thermometer for Sudbury granophyres and norite zircon samples, both analyzed for this project and compiled from literature^25,27^ compared with Jack Hills^39,55,56^, Acasta gneiss^40^, Bushveld^37^, and Barberton^58^ zircons, with results shown as PDFs. All Ti-in-zircon calculations assumed TiO_2_ and SiO_2_ activities of 1. **e** Th/U vs Yb ppm shows trends with different slopes for Sudbury norite and granophyre zircons. **f** Gd/Yb vs (Eu/Eu*)_CN_ shows that Sudbury granophyre zircons can have a more pronounced Eu anomaly than Sudbury norite zircons at higher Gd/Yb. These panels (**e**, **f**) are intended to highlight distinctions between zircons from the different layers of the melt sheet, although a version of this figure featuring comparison with early Earth zircons is available as Supplementary Fig. 15.

We compare REE concentrations of Sudbury melt sheet zircons with zircons from potential target rocks such as Levack gneiss or Mississagi quartzite, as well as zircons from early Earth environments. It is important to note that these comparisons for zircons are not 1 to 1 between the target lithologies and the rocks in the Sudbury melt sheet. Rather, zircon trace element chemistry in each lithology will be controlled by the trace elements in bulk source rock (e.g., measurements which for Sudbury can be found compiled in ref. ^34^), the zircon crystal-melt partition coefficient for each trace element, and by phases which crystallized prior to zircon in the melt and sequestered trace elements. Zircons from the melt sheet rocks generally have REE concentrations about the same to 5.5 times higher, excepting Ce, depending on the REE compared and whether granophyre or norite is being compared to Levack gneiss or Mississagi quartzite (Fig. 3a) zircon. Average Ce concentration from norite zircons is ~eight times the Levack gneiss zircon average. The ratio of granophyre zircon Eu concentration to the average Levack gneiss zircon Eu concentration is 0.7, or 0.6 if compared to Mississagi quartzite zircon. The average granophyre zircon Eu concentration is the only average REE concentration lower than the zircon average from a potential target rock. Zircons from the granophyre and norite layers have similar Heavy REE (HREE) concentrations (Fig. 3a), that is, from Gd to Lu, the ratio of the average granophyre zircon concentration divided by the average norite zircon concentration is around 0.9–1.3, depending on the element. Considering the three >3.2 Ga localities in this study, Jack Hills and Barberton have similar maximum measured concentrations, while Acasta gneiss can be 1.2–2.7 times higher from Gd to Yb, depending on REE (Fig. 3b). The range of measured zircon REE concentrations from the Bushveld igneous complex^37^ overlaps well with HREE concentrations from the Jack Hills. Minimum concentrations for the Sudbury granophyre zircons are within any of the three localities of interest, but the maximum possible REE concentration is higher for all REEs other than Eu.

Granophyre zircons tend to have larger calculated negative Eu anomalies than zircons from the quartz diorite or the norite samples, indicating either a more reduced region of the melt sheet or different co-crystallizing phases (Fig. 3c). Comparisons of Eu anomalies with the other zircon populations are discussed further in the supplemental. The Ti-in-zircon thermometer shows that the median crystallization temperature for the norite zircons is about ~90 °C higher than that of zircons from the granophyre. The median crystallization temperature of Jack Hills Hadean zircons (686 °C) is closest to the median value for the Acasta gneiss (681 °C) with the median value for Sudbury granophyre also close (664 °C) (Fig. 3d). The Jack Hills zircons may come from a more felsic melt^38^ so it is intriguing that that more felsic layer of the melt sheet shows more similar zircon crystallization temperatures. While there is some overlap, Fig. 2d indicates that the temperature PDFs show different populations.

Zircons from the Sudbury norite and granophyre layers can be distinguished via Th/U vs Yb ppm (Fig. 3e), and granophyre zircons can have a lower Eu anomaly at higher Gd/Yb (Fig. 3f). Geochemical differences for all these different zircon populations can be driven by differences in parent melt composition, crystallization temperature, and co-crystallizing phases. While the Sudbury melt sheet was likely an initial single parent melt (ref. ^13^), the melt sheet differentiated into the geochemically distinct norite and granophyre layers. The geochemistry of the Sudbury zircons will reflect their origin from an impact melt, the composition of the parent melt sheet, and finally the P–T and composition pathways that different regions of the melt sheet experienced as it differentiated. This last feature can be seen clearly in Fig. 3e, f, which shows geochemical differences for zircons from the norite and granophyre layers.

### Machine-learning analysis with zircons from impact melts

The “Methods” and Supplementary Information contain the data workflow (Supplementary Fig. 1), zircon filtering protocols to avoid inclusions, and performance metrics (e.g., accuracy, false positive rate, F1 score, etc.) for the SVM classifier. To test the utility of our approach in geoscience, we first applied SVMs to oceanic and continental zircons. This application produced similar results (Supplementary Fig. 17) to those previously published for oceanic and continental zircons^22^ and demonstrated using an SVM to combine three discriminant diagrams into one equation that distinguishes between these two zircon types.

We then trained a SVM on a compilation of zircons from I-, S-, A-type rocks^28^ to constitute an “endogenous zircons” classification, and Sudbury impact melt zircons as the “exogenous zircons” classification. The I-, S-, and A- rocks, “endogenous zircons”, compilation featured *n* = 3794 zircons compiled from other publications and was filtered by La <1 ppm along with the Light Rare Earth Element-Index (LREE-I) > 30 from ref. ^39^. After further filtering, a total of 3369 endogenous zircon analyses were input to the SVM. The Sudbury melt sheet zircons, which total *n* = 328 used by the SVM after filtering, using the same La <1 ppm and LREE-I > 30 conditions of ref. ^28^, are primarily from this work but include an additional *n* = 24 zircon analyses from ref. ^25^. We used element and ratio inputs of U/Yb, Th/U, Yb, and Gd/Yb; reasons for selecting each are covered in the methods and the supplemental material. The SVM achieved an accuracy of 79% on Sudbury zircons withheld from the training set as a test for model accuracy and 98% on withheld endogenous zircons, and a F1 score of 0.79.

After model training, we evaluated Acasta Gneiss Complex^40,41^, Barberton Greenstone Belt^37^, and Hadean Jack Hills zircons (Table 1). Our SVM did not identify more than 1 zircon as exogenous. Since the holdout accuracy is ~80% on impact melt sheet zircons, the identification of a single grain as exogenous is not significant. Therefore, if these early Earth zircons formed from impact melts, their chemistry differs from that of the Sudbury impact melt zircons used to train the model.

**Table 1:** Identification of Early Earth zircons as exogenous or endogenous by support vector machine

Zircon identification with SVM1, Kernel: rbf, C = 1000, ɣ=1, no class balancing	% Sudbury - as “exogenous zircons” archetype	% I-, S-, A- - as “endogenous zircons” archetype
Jack Hills zircon (*n* = 97)	1% (*n* = 1)	99% (*n* = 96)
Barberton Greenstone Belt (*n* = 87)	0% (*n* = 0)	100% (*n* = 87)
Acasta Gneiss zircon (*n* = 92)	0% (*n* = 0)	100% (*n* = 92)

## Discussion

One consideration is whether a Hadean impact melt is similar to the 1.85 Ga Sudbury impact melt. While the rock types of the target material could be different, there are key similarities (e.g., the compositional similarities of the Sudbury target and TTGs suggested by Fig. 2). In addition, assuming that the temperatures reached in most hypervelocity impacts are high enough, the impact melts produced will have homogenized target materials well above the liquidus of most materials. After the melt, it will differentiate as it cools. Given the volume of differentiated melt sheet mass for Sudbury, the Sudbury melt sheet is analogous to larger melt sheets that were produced on the early Earth and also underwent differentiation. Given the paucity of Hadean zircon samples, it is reasonable to assume that if surviving Hadean zircons were from an impact melt sheet, they were from a larger impact event that experienced differentiation like Sudbury. Therefore, given the target rock compositional similarities (Fig. 2) and our consideration that if Hadean zircons were sourced from an impact melt sheet, it was a larger one, we considered Sudbury a good analog for impacts capable of crystallizing Hadean zircons.

The Sudbury event was an impact into continental crust^42^ although the precise depth of melted material has been a subject of ongoing study. Impact modeling of the Sudbury structure reconstructs a transient cavity diameter of ~100 km (ref. ^43^), which implies a melt zone extending to a depth of ~30 km (ref. ^44^). This implies that the melt sheet is derived from a column of both upper and middle crust, rather than solely from surface supracrustal rocks. In addition, the melt sheet chemistry may be derived from a lower crustal^45^ component while no mantle component is required to match the geochemistry^46^. Prior research^24^ indicates that almost all impact-related zircons plot in the continental field on the diagrams in ref. ^22^. Consequently, this geochemical signature could be common to impact melting. Still, one possible reason we did not detect zircons with impact geochemistry on the early Earth is that impacts into oceanic crust result in zircons with notably different compositions. If interpreted this way, then we do not see zircons with Sudbury-like impact melt geochemistry on the early Earth because significant quantities of continental crust to impact did not exist. Instead, the early planet was mostly oceanic crust. Likewise, while impacts into Hadean mafic crust or tonalite–trondhjemite–granodiorite (TTG) rocks could result in zircons different from the Sudbury zircons used to train our exogenous classification, our petrologic analysis (Fig. 2) implies some similarity between the target rocks at Sudbury and Archean TTGs. Our results are thus a strong indicator that the early Earth zircons analyzed are not sourced from impacts into continental crust-like material, especially anywhere the target rock geochemistry is like that at Sudbury.

Another way of tracing historical impact events is via mineral shock microstructures. While minerals that crystallize in-situ in a melt sheet will be unshocked, excepting additional impacts, ejecta and nearby minerals may record physical evidence of the original event. Research on shocked zircons from Vredefort has implied that shocked microstructures will survive in the geologic record^47^, but these microstructures are yet to be detected in Hadean or early Earth zircon grains. So, currently, the available early Earth detrital zircon record provides no evidence for continent-derived impact melt zircons or shocked zircons.

**Fig. 4:**
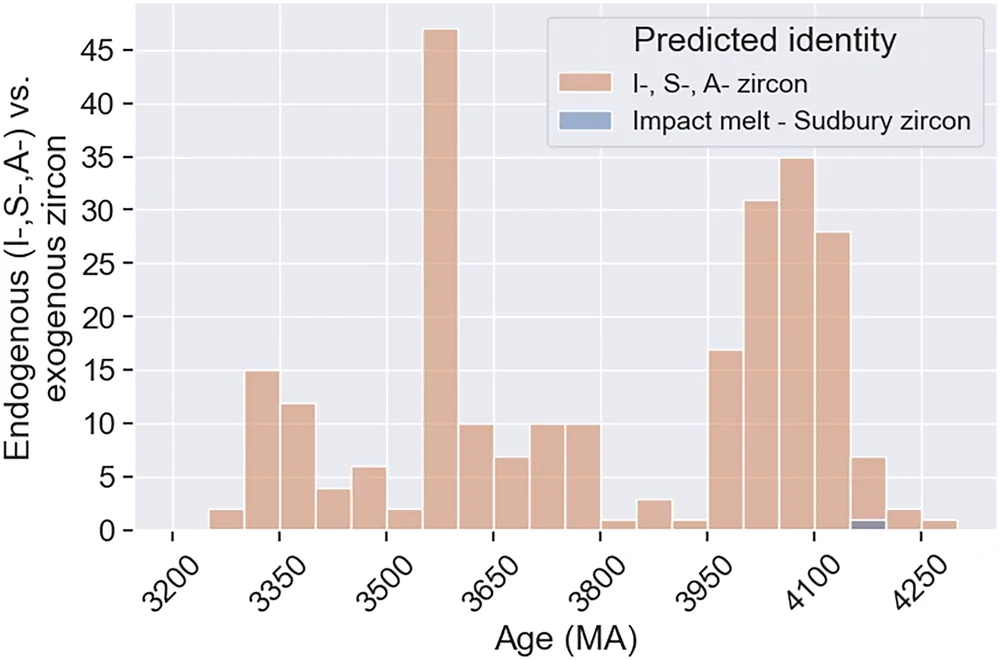
Age distribution of the >3.2 Ga zircons analyzed, indicating whether the SVM indexed these zircons as endogenous or exogenous.

These results suggest lower impact frequency for the early Earth from the perspective of the preserved zircon record. Impact frequency moderates early planetary habitability by influencing whether oceans can survive or are continuously vaporized^48^, which is particularly important for photosynthetic organisms. Hadean zircon chemistry supports the presence of a pre-4.0 Ga hydrosphere^15,49,50^, implying a more habitable Hadean. Our results indicates no evidence for a late heavy bombardment, since there is no 4.1 Ga cluster of impact melt sheet zircons (Fig. 4). Thus, our analysis implies endogenous melting as the more probable source for zircons from the >3.2 Ga localities studied, that the Hadean and early Earth zircons are not sourced from impacts into continental crust-like material, and that a Hadean Earth hydrosphere unperturbed by many impacts is plausible.

## Methods

### Zircon mineral separation, zircon imaging, and LA-ICP-MS analysis

We collected samples from the Sudbury melt sheet to populate a “zircons from impact melt sheet” class (Fig. 1). Zircons were extracted by crushing the rocks via a jaw crusher and disk mill and sieving this material to x < 300 μm grain fractions. These grain fractions were then magnetically sorted via a Frantz with at least 1 pass at 0.3 A and another pass at 1.2 A. The non-magnetic grain fractions were sorted by density using lithium metatungstate (LMT). Then, from the non-magnetic heavy grain fractions, zircons were picked and mounted in epoxy. Mounts were polished with 600-grit sandpaper and finished using an automated polisher and 1 μm alumina powder. Our original granophyre samples from the north range did not yield zircons, so we supplemented our collection with additional mineral separates used in ref. ^25^ from which we were able to acquire additional analyses to populate the granophyre classification. These granophyre samples were also from the south range.

Samples were imaged at the University of Rochester with a Hitachi TM-2000 scanning electron microscope (SEM) at an accelerating voltage of 15 kV. Some zircons were additionally imaged via backscatter electron imaging (BSE) and cathodoluminescence (CL) at the Smithsonian Institution. We quantified zircon trace elements using a Photon Machines Analyte G2 laser ablation (LA), attached to an Agilent 7900 inductively coupled plasma (ICP-MS) mass spectrometer at the University of Rochester. A 20-μm spot was used, with a rep rate of 10 Hz, a fluence of 4.72 J/cm^2^, and a shot count of 150. Washout times were at least 55 s or longer between each analysis. Isotopes analyzed were ^23^Na, ^29^Si, ^45^Sc, ^49^Ti, ^89^Y, ^91^Zr, ^93^Nb, ^139^La, ^140^Ce, ^141^Pr, ^146^Nd, ^147^Sm, ^153^Eu, ^157^Gd, ^159^Tb, ^163^Dy, ^166^Er, ^172^Yb, ^175^Lu, ^206^Pb, ^207^Pb, ^232^Th, ^238^U, although some sessions did not include ^91^Zr. Analyses were reduced using Iolite 3 (ref. ^51^) with the X_Trace_Elements_IS DRS^52^ for trace elements, with ^29^Si was used as the internal standard at a wt% level of 14.8, and X_U_Pb_Geochron4 DRS for ages and Pb isotopes^53^ to check that impact melt sheet zircons, broadly, matched the age of the impact. Standards were NIST 612 for the trace element analyses or hand-picked Duluth gabbro zircon for the Pb and geochronology analyses. We screened zircon with high ^23^Na counts, which were excluded from further consideration. Finally, all analyses with La concentration greater than 1 ppm were removed from consideration. Overall, these considerations resulted in filtering out between ~7% to nearly 90% of the analyses, depending on the source rock. A discussion of the types of mineral inclusions present, depending on the source rock, is available in the supplementary, along with SEM images with EDS spectra.

### Further zircon analyses and literature training data sources

The total dataset of impact melt zircons' geochemistry is not large. For example, impact melt sheet zircons from three studies include *n* = 145 from ref. ^27^, *n* = 136 from ref. ^25^, and *n* = 86 from different melt sheets^24^ totaling 367. In addition to limited samples, a further complication arises from the nature of geochemical datasets, particularly when they are aggregated from diverse sources. Many machine-learning algorithms require fully populated input features; as a result, missing values must be handled through interpolation or imputation to ensure the data remains usable, or the analysis must be discarded. Therefore, we present several additional Sudbury zircon trace elements analyses totaling *n* = 304 from the main mass with this study. We also relied on published zircon analyses to both supplement our own analyses, populate an endogenous classification in training the SVM, and have pre 3.2 Ga zircons to predict on. We compiled Supplementary Data 1 based on our analyses of zircons from the Sudbury impact melt sheet and combined it with available literature analyses. As in all geological investigations, one must be aware of sampling and preservation biases. Since this work used zircons, a mineral very robust to chemical and physical weathering^54^, preservation biases are minimized. Further, we justify our use of 1.85 Ga Sudbury impact as the best available analog for even older impacts on the early Earth. As acknowledged throughout the paper, the Sudbury melt sheet represents an impact on continental crust material, and many of the target rocks have geochemical similarities to TTGs (Fig. 2) and rocks that existed on the early Earth.

Thus, we compiled our Sudbury zircon analyses with other Sudbury zircon datasets^25,27^. Then, to have a broad classification representing common endogenously derived zircons on Earth, we needed a dataset of zircons from I-, S-, and A-type granitoids. For this, we used a compiled I-, S-, A-, zircon dataset^28^ to populate a combined I-, S-, A-, class representing common endogenous Earth features. For the pre-3.2 Ga zircons we predicted on, we used Hadean Jack Hills zircons (*n* = 97) datasets^39,55–57^, the detrital zircons from the Green Sandstone Bed of the Barberton Greenstone Belt, *n* = 87 from ref. ^58^, and zircons from the Acasta Gneiss (*n* = 92) from ref. ^40^ comprising three separate localities. Rocks from the Acasta Gneiss Complex, specifically the 4.02  Ga Idiwhaa gneisses, may represent impact-derived melts based on evidence indicating their formation through partial melting at low pressures^9^. Although not a pre-3.2 Ga terrain, we also incorporated Bushveld zircons (*n* = 79) from ref. ^37^ into our REE plots and used them for SVM predictions in the supplementary information as well (Supplementary Table 3).

### Machine-learning training

Supplementary Tables 1 and 2 present the total number of data points for each rock after filtering in each group. Before applying any machine learning, we preprocessed the data by applying log_10_ to all analyses, to homogenize the scale of various features and avoid outsized effects of large values for one variable during training. To ensure that the model performs well on unseen data, we split the original data (i.e., the endogenous zircons and Sudbury impact melt sheet zircons filtered from Supplementary Data 1) into “training data” and “test data” in an 80–20 split, a commonly used ratio for this purpose^59^. Data was split by taking 80% of the original data at random for the training data and using the remaining 20% for the test data, such that there is no data leakage between the training and test data. The training data are used for training the model, while the test data are used to evaluate the performance of the model on previously unseen data. Given the imbalance between the large number of I-, S-, A- zircons and Sudbury zircons, we trained two SVMs. The first used no class balancing; in this case, 80% of each classification was selected as training data. This is SVM1 present in the main text. For the second model, we trained an additional SVM model using the training data from the 80–20 train-test split per class, where we then balanced classes to 1000 samples per class using oversampling and undersampling. The class-balanced model is covered further in the supplementary materials.

In addition, since many ML algorithms (including SVM) require complete input features (or interpolation of values), it is essential to select features that are most likely to be related to the scientific question without limiting the size of the data pool. In other words, including a rarely analyzed input will reduce the available data for training. The zircon features we selected were U/Yb, Th/U, Yb, and Gd/Yb, since each of these has been shown to possess valuable geological information for a zircon or the melt from which it originated. For example, the U/Yb ratio is related to the input of continental or oceanic crust^22^. Meanwhile, Th/U can be related to crystallization behavior, such as whether the zircon crystallized in equilibrium or disequilibrium with the melt^60^. The concentration of Yb is a proxy for the HREEs in the source melt. Finally, the Gd/Yb ratio measures medium-REE (MREE) enrichment and can reflect garnet growth under specific melting conditions^23,61^. After training, we used the model to predict on the test set to evaluate its accuracy on new, unseen data of the same classifications.

Most machine-learning algorithms (including SVMs) have hyperparameters, which can be tuned to adjust how the machine-learning algorithm works and can affect the performance of the model^62^. The key parameters for SVMs are the kernel, *C* (the regularization parameter), and, only for specific kernels, the gamma parameter. The regularization parameter *C* controls how much the model penalizes misclassified data points and can be interpreted as controlling how much “slack” is allowed in the fit. When using a linear kernel, *C* is the only hyperparameter to adjust. In the more complex radial basis function (RBF) kernel, both *C* and gamma can be varied. The gamma parameter determines the curvature allowed in the decision boundary, with higher gamma allowing more curvature. These parameters (kernel, *C*, and gamma) were fit using a grid search procedure detailed further in the supplementary information.

### Machine-learning evaluation and predictions

We evaluated the performance of the SVM models on the test set by calculating the accuracy per class. Accuracy is defined as the number of correct predictions for each class divided by the total number of members in that class. We also calculated the false positive rate, false discovery rate, and *F*_*1*_ score. The *F*_*1*_ score is a balanced accuracy metric, with better-performing models scoring closer to 1. Calculating the *F*_*1*_ score for a binary classifier is sensitive to which classification is labeled as “positive”; in our case, this was set as the impact melt zircon. To address this sensitivity, we also calculated the P4 metric, which resolves the issue. Both calculations favored SVM1. Finally, the models were applied to early Earth zircons to evaluate how well the models classified them. The early Earth zircon analyses used for predictions were prepared using the same data preparation (La <1 ppm and LREE-I > 30) and transformations (taking log_10_) on each feature (i.e., U/Yb, Th/U, Yb, Gd/Yb) as was done for the training data and test data.

## Supplementary information

Supplementary Information

Description of Additional Supplementary Files

Supplementary Data 1

Supplementary Data 2

Supplementary Data 3

Transparent Peer Review file

## Data Availability

Much of the data used is already available from various literature sources. Data necessary to run the machine-learning algorithm has been compiled together in an easy-to-parse format available as Supplementary Data 1 for the main text. The zircon analyses used for the machine-learning analysis of oceanic and continental zircons in the supplementary information file are compiled into a file titled Supplementary Data 2. The whole rock analyses from the literature from Sudbury whole rocks and from TTGs used to make Fig. 2 are available as Supplementary Data 3.

## Code availability

The code used for this project’s analysis is available on the Code Ocean platform at https://doi.org/10.24433/CO.6637839.v1. Running the Jupyter notebook on the Code Ocean platform will generate the plots (before stitching into a composite figure) and analysis used for this manuscript.
