# Supplementary material for: A missing continental impact melt sheet signal in the early Earth detrital zircon record
Source: Nat Commun. 2026 Aug 11;17:10106. doi: 10.1038/s41467-026-76529-w (PMC13598112; doi:10.1038/s41467-026-76529-w)
Supplement: Supplementary file 1 [file 41467_2026_76529_MOESM2_ESM.pdf]

Description of Additional supplementary files

Title: Supplementary Data 1

Description: "Compilation of new zircon analyses from the Sudbury impact structure from this project along with literature zircon analyses from Sudbury, endogenous I-,S-,A- zircons, and early Earth zircons. Used for machine learning analysis in the main text.”

Title: Supplementary Data 2

Description: "Compilation of trace element oceanic and continental zircon analyses from the literature. Used for machine learning analysis in the supplementary information to refine the oceanic and continental zircon discriminant diagrams."

Title: Supplementary Data 3

Description: "Whole rock analyses from the literature used to produce Figure 2. Compilation of whole rock analyses of Archean tonalite-trondhjemite-granodiorite (TTG) rocks, Sudbury melt sheet rocks, and Sudbury target rocks such as Levack gneiss. "
