# Supplementary material for: A missing continental impact melt sheet signal in the early Earth detrital zircon record
Source: Nat Commun. 2026 Aug 11;17:10106. doi: 10.1038/s41467-026-76529-w (PMC13598112; doi:10.1038/s41467-026-76529-w)
Supplement: Supplementary file 2 [file 41467_2026_76529_MOESM6_ESM.pdf]

nature portfolio

Peer Review File

A Missing Continental Impact Melt Sheet Signal in the Early Earth Detrital Zircon Record

Corresponding Author: Dr Ian Szumila

This file contains all reviewer reports in order by version, followed by all author rebuttals in order by version.

Version 0:

Reviewer comments:

Reviewer #1

(Remarks to the Author)
The manuscript by Szumila et al. use trace element characteristics of zircon from the Sudbury Igneous Complex as an analogue for early Earth impact processes. The data and machine-learning approach appear to be robust. I think a greater effort needs to be made to show that the geological setting (e.g. rock types) prior to the Sudbury impact is analogous to an Early Earth setting. In particular, the rock types in Figure 1 are rare to absent in the Eoarchean and Hadean. Because the primary driver of trace element compositions in zircon is that of the melt from which it crystallizes (and the melt is produced from the rocks near the surface during impact), I think the authors should explore the similarities (and differences) in more detail before applying the classification of Sudbury-related zircon to much older rocks that were likely quite different. More fundamentally, why would an impact change the U/Yb, Th/U, and Gd/Yb contents of the melt from which a zircon crystallizes?

If indeed there are differences in trace element signatures of zircon between impact and non-impact related system, what are the underlying petrological drivers that yield a difference? The machine-learning approach points one in a direction that there could be a difference (I see this from the data), but this should always be followed up with a petrological/geochemical reason why there is a difference and then expanded to other questions (such as impacts on Early Earth).

Comments by line number:

L52: One can argue that the pre-existing lithologies before impact (including abundant metasedimentary material) were rare to absent in the Hadean. See the various rock types in Figure 1 that underly the SIC, which includes carbonates. Why then is this melt sheet analogous to those that could have existed in the Hadean?

L63: Zircon only retain parent melt composition if they have not been recrystallized or subject to later metamorphism, which is not always the case with Hadean zircon.

L63: "Can distinguish" is too strong for something that works only some of the time. I suggest toning this down.

L80: Again, machine learning-derived classifications can provide a probable source, but there are always exceptions. I suggest rephrasing with this caveat.

L87: This comparison implies that trace elements in zircon in the target rocks would be the source of trace element signatures in zircon that crystallized from melt. However, the zircon are a very minor part of the rocks that underwent melting during the Sudbury impact event. Trace element signatures in zircon from crystallization of the melt sheet would mainly be from the dominant silicate minerals in the target rocks, not from the zircon.

L117: How much more reducing would you expect this to be and is this in line with the estimates and models of fO2 in the different rock types (e.g., norite, granophyre) during crystallization? (see Ariskin et al., 1999, GeoSocAm Speical Paper 339, p373-387). Could it be that there was a fair amount of organic material (e.g. from the sedimentary rocks impacted) that led to a very reducing system and very negative Eu anomalies? Although this is not crucial for your study, this implies that there are many unknowns about the evolution of the melt sheet. If that is the case, why is it used as the type example to relate to Hadean zircon?

L119: I wouldn't say that the difference in Eu anomalies is discussed in the supplement. It is described. I see no interpretations (and discussion implies interpretations).

L127: Yet most argue that the Sudbury melt sheet started as a single parental melt. Therriault et al., 2002, Econ Geol, 1521-1540. So, what is your evidence that the parent melt was variable?

L139: Considering the notorious problem with lots of laboratories measuring quantifiable La concentrations in zircon, can you specify why you chose a La < 1 ppm threshold?

L157: I disagree. The underlying rock types were probably quite different (as I mentioned earlier as a comment on L52).

L160: Good analogues require similar boundary and starting conditions. The dominant control on trace elements in zircon in any igneous system is the parental melt. Can you justify that the parental melt compositions for the SIC and the Hadean melt sheets were similar? If not, can you at least infer that the crystallization sequences would be similar so that the imprint of various minerals you mention in your manuscript that control the investigated ratios (e.g. garnet on L271).

L174: I don't follow the 'even then' logic in this sentence. If you train your algorithm on a different case study (like a TTG), then would the results not be different? And many studies of early Earth (Hadean and Eoarchean) argue that the upper continental crust (susceptible to an impact) was more mafic. TTGs are usually emplaced in the middle crust (and may or may not have participated). This would have implications for the U/Yb, Th/U, and Gd/Yb of the parental melt sheet, the crystallization sequence, and the resultant composition of zircon that crystallized from this magmatic system. So, is this really analogous to the Sudbury scenario? Perhaps I am missing a link here, but this sentence (which is a key link in this manuscript) seems to mislead the reader and suggest equivalence between the early Earth and the Sudbury example regardless of the fundamental geological differences in rock types.

(Remarks on code availability)

Reviewer #2

(Remarks to the Author)
Overall this is a welcome and thorough investigation using high quality geochemical inputs and a novel machine-learning application to address a longstanding debate. The authors have extrapolated their model, trained on the trace element composition of Sudbury melt sheet zircons, to test for evidence of Hadean melt sheets (targets unknown). It is proposed that the negative outcome of their test indicates that the Jack Hills Zircon suite is not impact-derived. I found this to be a bit of an overreach. As the authors acknowledge in L168-175, their Sudbury training model does not capture zircons produced by melt sheets derived from OCEANIC crust. They are correct that their model is best suited to impact melting of continental crust; "Even then, our results indicate that the zircons analyzed are not sourced from impacts into continental crust like material.L174". So, I support publication of this work, but with a less expansive conclusion and title along the lines of "No Evidence for Continent-derived Impact Melt Sheets from the Early Earth Detrital Zircon Record". This more limited implication is not the authors' fault, as there are no easily accessible melt sheet zircons from oceanic crust targets available for training, to the best of my knowledge...the Mjolnir impact in North Sea is perhaps the best potential target but drill cores from the Norwegians are not easily accessed. In the absence of natural samples, or a modelled data set for zircon chemistries from ocean-crust-derived impact melting, the authors might consider limiting the scope of their conclusions. Otherwise it is a sound and valuable contribution that is more rigorous than most other recent examinations of Jack Hills zircon geochemistry in this light.

(Remarks on code availability)

Open Access This Peer Review File is licensed under a Creative Commons Attribution 4.0 International License, which permits use, sharing, adaptation, distribution and reproduction in any medium or format, as long as you give appropriate credit to the original author(s) and the source, provide a link to the Creative Commons license, and indicate if changes were made.
In cases where reviewers are anonymous, credit should be given to 'Anonymous Referee' and the source.
The images or other third party material in this Peer Review File are included in the article's Creative Commons license, unless indicated otherwise in a credit line to the material. If material is not included in the article's Creative Commons license and your intended use is not permitted by statutory regulation or exceeds the permitted use, you will need to obtain permission directly from the copyright holder.
To view a copy of this license, visit https://creativecommons.org/licenses/by/4.0/

We thank the editor for their handling of our manuscript and the reviewers for their comments. Below we address point by point the remarks by the reviewers. We have also broken up the first paragraph of the first review so that our responses will be more direct to the points raised there. References cited in this response (including references for quoted text from the manuscript or supplemental) are in their own bibliography at the bottom of this document.

REVIEWER COMMENTS

Reviewer #1 (Remarks to the Author):

The manuscript by Szumila et al. use trace element characteristics of zircon from the Sudbury Igneous Complex as an analogue for early Earth impact processes. The data and machine-learning approach appear to be robust. I think a greater effort needs to be made to show that the geological setting (e.g. rock types) prior to the Sudbury impact is analogous to an Early Earth setting. In particular, the rock types in Figure 1 are rare to absent in the Eoarchean and Hadean. …

We appreciate the reviewer's comments regarding the target lithologies and have revised the text to more clearly define why the Sudbury Igneous Complex (SIC) remains the most robust terrestrial analogue for Hadean impact processes. We emphasize that the primary contributor to the SIC melt sheet by volume was the underlying crystalline basement rather than the sedimentary cover. As summarized by ref. [1], the North Range footwall consists of the Levack Gneiss Complex, a 2.7 Ga suite dominated by tonalitic to granodioritic gneisses and migmatites. These Archean granitoids are of sodic, silica-rich, infracrustal character that serves as a reasonable proxy for the tonalite trondhjemite granodiorite (TTG) crust thought to be common in the Eoarchean and Hadean. To illustrate this more directly we have now included a comparison of major element oxide compositions of the Sudbury target rocks to show that they overlap directly with Archean TTGs (See the new **figure 2** in the main text).

While the Huronian units are Proterozoic and sedimentary, their inclusion in the model does not imply a direct stratigraphic equivalence to Hadean materials. Rather, they represent the incorporation of supracrustal sequences into an impact melt. This is consistent with evidence from Hadean zircon suites, where isotopic signatures have been interpreted to reflect the remelting of weathered or sedimentary protoliths. We argue that the combination of a massive impact melt sheet, a well-characterized TTG-like basement (see new **Figure 2**), and a subordinate supracrustal sequence makes the Sudbury target rocks the best available analogue on Earth for a large-scale Hadean impact event. We

have also updated the caption to the Sudbury map (**Figure 1**) to clarify this.

…Because the primary driver of trace element compositions in zircon is that of the melt from which it crystallizes (and the melt is produced from the rocks near the surface during impact),…

We would like to take a moment to clarify the extent of melting. The reviewer notes that the melt is produced from rocks 'near the surface.', to which we add that impact modeling of the Sudbury structure reconstructs a transient cavity diameter of ~100 km (ref. [2]) which implies a melt zone extending to a depth of ~30 km (ref. [3]). This implies that the melt sheet is derived from a column of both upper and middle crust, rather than solely from surface supracrustal rocks. We make this point now more explicitly in the discussion with the following revision to the main text:

"The Sudbury event was an impact into the continental crust[4] although the precise depth of melted material has been a subject of ongoing study. Impact modeling of the Sudbury structure reconstructs a transient cavity diameter of ~100 km (ref. [2]) which implies a melt zone extending to a depth of ~30 km (ref. [3]). This implies that the melt sheet is derived from a column of both upper and middle crust, rather than solely from surface supracrustal rocks."

…I think the authors should explore the similarities (and differences) in more detail before applying the classification of Sudbury-related zircon to much older rocks that were likely quite different.…

We emphasize that many trace element concentrations and ratios could, in practice, be incorporated into our support vector machine (SVM) algorithm. We selected the specific parameters used here because they have demonstrated strong diagnostic value in prior studies, in some cases for nearly two decades. Building on this foundation, we leverage well-established trace element signatures derived from terrestrial melting environments and extend their application to terrains of different origin, specifically impact-generated systems. At the same time, we expand the methodological framework by applying a support vector machine approach to evaluate and classify these geochemical signals.

These geochemical ratios were picked due to their utility in previous studies. Particularly in ref. [5],three plots of U vs Yb, U/Yb vs Hf and U/Yb vs Y were used to build discriminant diagrams that identified regions where zircons tended to plot if they were from oceanic crust or continental crust. In the later work, i.e. ref. [6], more of the mechanism behind these the concentrations of elements ratios is detailed. For example, increased U/Yb is associated with either crustal input or more enriched mantle sources (Figure 1 from ref. [6]). Changes in Gd/Yb measure MREE enrichment and are also related to garnet

crystallization in the same melt, as well as titanite and apatite fractionation. The Th/U ratio in a zircon can indicate variations in the magma during crystallization (ref. [7]). Importantly, similar ratios were utilized in the work of ref. [8] which plotted impact zircons on the same three discriminant diagrams of ref. [5] (i.e. of U vs Yb, U/Yb vs Hf and U/Yb vs Y). They found that impact melt zircons, from Sudbury, Morokweng, Manicouagan, and Vredefort, plot almost exclusively in the continental region originally defined by ref. [5].

We chose to train our machine learning system on U/Yb, Th/U, Gd/Yb and Yb with zircons from I-, S-, A- granites as our endogenous case and zircons from the Sudbury impact melt sheet as our representative of an impact melt sheet classification. One key idea behind this work is that we expected impact melts could have different geochemical signatures than endogenous processes. Our reasoning was that an impact event involves a producing a new amalgamated melt of many different rock types which does not mimic endogenous melting processes. To address these points we have now added a new section in the supplementary: "**Petrologic Rationale for Feature Selection**"

…More fundamentally, why would an impact change the U/Yb, Th/U, and Gd/Yb contents of the melt from which a zircon crystallizes?

This is an excellent question and goes to the core of what we are evaluating. On Earth, most melting processes follow relatively well understood thermodynamic paths. For example, mantle melting during adiabatic decompression produces partial melts that commonly display systematic enrichment of the light rare earth elements relative to the heavy rare earth elements. Although the absolute pattern depends on factors such as melt fraction and whether the mantle source is enriched or depleted, the resulting signatures are regular and reproducible.

Crustal melting processes, while more complex, behave similarly. Anatexis or partial melting of granitic protoliths also generates predictable trace element fractionations, including light-to-heavy rare earth element enrichment patterns and systematic behavior among elements such as U, Th, and Hf.

In contrast, an impact melt sheet forms through rapid, heterogeneous melting of mixtures that can include sediments, basement lithologies, and other target materials. This process is stochastic and is not governed by the equilibrium or near-equilibrium pathways typical of endogenic melting. We therefore hypothesize that the resulting melts, and the minerals that crystallize from them, will record trace element signatures that differ from those produced by conventional terrestrial melt-generation mechanisms. If correct, these signatures may preserve information about the origin and nature of the impact-derived melt. Also see additions to the new supplementary section: "**Petrologic Rationale for Feature Selection**"

If indeed there are differences in trace element signatures of zircon between impact and non-impact related system, what are the underlying petrological drivers that yield a difference? The machine-learning approach points one in a direction that there could be a difference (I see this from the data), but this should always be followed up with a petrological/geochemical reason why there is a difference and then expanded to other questions (such as impacts on Early Earth).

Yes, we do detect differences in the trace element signatures of zircons between the Sudbury impact and the non-impact related systems for which they are compared. And with respect to the underlying petrological drivers that yield a difference, please see the comment above on the differences we expect between crustal melting and impact melting. We agree that it's important to follow this up with a petrological and geochemical reason for why this difference exists and have emphasized this in the text of the manuscript and supplemental. We think these differences are likely driven by Sudbury being a melt of primarily crustal material, that produced a homogenous melt sheet (refs. [9,10]) that then differentiated. The temperatures experienced in the impact would have also caused extensive degassing (refs. [11,12]), leading both to the loss of volatiles and playing a role in the formation of the ore deposits.

Comments by line number:

L52: One can argue that the pre-existing lithologies before impact (including abundant metasedimentary material) were rare to absent in the Hadean. See the various rock types in Figure 1 that underly the SIC, which includes carbonates. Why then is this melt sheet analogous to those that could have existed in the Hadean?

Although the relative proportions of lithologies at Sudbury Basin are unlikely to reproduce the full stochastic diversity of impacts that must have occurred on the Hadean Earth, they remain highly relevant. Oxygen isotope data from Hadean zircons provide strong evidence that metasedimentary materials were present at Earth's surface and were incorporated into magmatic systems (refs. [13,14,15]). Thus, inclusion of sedimentary components in the target assemblage is not only plausible but expected.

Furthermore, the presence of liquid water under a fainter young Sun implies elevated atmospheric CO_2 relative to the modern Earth. Over time, silicate weathering would have drawn down this CO_2, promoting the formation and accumulation of carbonate-bearing lithologies at the surface. Their potential involvement in impact melting scenarios is therefore consistent with first-order models of early Earth surface environments. We reiterate an additional key point: geochemical and isotopic constraints indicate that a substantial fraction of the melt sheet was derived from crystalline basement.

This contribution includes rocks of the Levack Gneiss Complex, which dominate the target by volume and provide an appropriate analogue for evolved Archean continental crust.

Therefore, although Sudbury is an impact occurring at 1.85 Ga, it is a strong analogue for an impact into Hadean continental crust material. Additionally, it is the largest and best preserved melt sheet on Earth. Given the inability to study Hadean impacts directly and the lack of preserved Hadean rocks, the older age, and accessibility of the Sudbury Igneous Complex makes this is currently our best chance at building a broad impact melt zircon geochemistry dataset which is a necessity for the application of machine learning.

L63: Zircon only retain parent melt composition if they have not been recrystallized or subject to later metamorphism, which is not always the case with Hadean zircon.

We changed language on this line to ‘help to constrain’ to acknowledge the later metamorphism or recrystallization cases and other possible limitations in the general case. While zircon commonly preserves trace element signatures inherited from its parent melt, several key considerations are important in evaluating this comment. If zircon were to recrystallize, the process would be expected to expel Pb, which is highly incompatible in the zircon structure, thereby resetting the U–Pb system. Preservation of primary ages therefore argues against wholesale recrystallization.

In addition, modification of trace element compositions in zircons requires metamorphic conditions far more extreme than those needed to disturb trace element systems in most other minerals. Diffusive length scales for most trace elements in zircon are exceedingly small, meaning that substantial re-equilibration is difficult to achieve without near-complete dissolution and regrowth. Consequently, the trace element signatures we use to infer parent melt composition are generally robust to anything short of very high-grade metamorphism. For example, even at 1000 °C sustained for 100,000 years, predicted diffusion distances are on the order of micrometers or less: ~1–2 μm for Yb (ref. [16]) and only nanometer-scale for Th, U, and Hf (ref. [17]). Such distances are negligible relative to typical zircon growth zoning, reinforcing that preservation of primary trace element patterns is expected unless the crystal undergoes wholesale recrystallization.

L63: "Can distinguish" is too strong for something that works only some of the time. I suggest toning this down.

We agree with this statement and toned this down by changing ‘can distinguish’ to ‘can be used to infer’.

L80: Again, machine learning-derived classifications can provide a probable source, but there are always exceptions. I suggest rephrasing with this caveat.

We agree with this statement and caveat. The relevant line has been changed to:

"Differences between I- and S-type granites were first recognized in the Lachlan fold belt[32] and current machine learning methods can successfully use trace element measurements to determine whether a zircon originated from an I-, S-, or A-type granite[31] with accuracies greater than 85%."

The Zhong et al., 2023 paper referenced here, trained multiple ML classifiers including support vector machine, random forest, and multi-layer perceptions with the resulting accuracies in identifying whether zircons were from an I-, S-, A-type granite being between 86% and 89%. The line has been revised to mention this result and accuracy.

L87: This comparison implies that trace elements in zircon in the target rocks would be the source of trace element signatures in zircon that crystallized from melt. However, the zircon are a very minor part of the rocks that underwent melting during the Sudbury impact event. Trace element signatures in zircon from crystallization of the melt sheet would mainly be from the dominant silicate minerals in the target rocks, not from the zircon.

This implication is not intended from this comparison. This study is primarily focused on differences between zircons from I-, S-, A- granites and those from the Sudbury melt sheet and then using a machine learning model trained on these types to classify early Earth zircons. We have added a reference to whole rock compositions as reported in ref. [1] to avoid this section giving the implication that the melt sheet zircons and target rock zircons are 1-to-1 comparable without considering their source whole rocks. Additionally, we have made plots using the reported whole rock concentrations from Lightfoot et al., 2016 and added them to the supplemental.

Due to our field work at Sudbury, we had the means to collect rocks from potential target rock (Levack gneiss, Mississagi quartzite) as well as later infilling sediment (Chelmsford turbidite) and we measured the trace elements of zircons in these rocks. We wanted to also report these results and felt they also helped contextualize the trace element measurements in zircons generally. We agree with the reviewer. The zircon trace element signature would come primarily from the overall composition of the melt sheet or from the melting and differentiation process and not from the zircon trace element contents of target rocks.

L117: How much more reducing would you expect this to be and is this in line with the

estimates and models of fO2 in the different rock types (e.g., norite, granophyre) during crystallization? (see Ariskin et al., 1999, GeoSocAm Speical Paper 339, p373-387). Could it be that there was a fair amount of organic material (e.g. from the sedimentary rocks impacted) that led to a very reducing system and very negative Eu anomalies? Although this is not crucial for your study, this implies that there are many unknowns about the evolution of the melt sheet. If that is the case, why is it used as the type example to relate to Hadean zircon?

In ref. [18], two different potential parent melts (named SIC and QRN) for Sudbury are simulated with the COMAGMAT software. While the Eu contents of these simulated melts is not reported directly in ref. [18], Figure 10 of that work shows an REE diagram of the granophyre and quartz-gabbro rock normalized to mafic norite based on the data of Ostermann, 1966. Ref. [18] found that the proposed SIC parent melt composition was relatively independent of fO_2. The proposed QRN parental melt is more dependency on fO_2 due to the crystallization temperature of magnetite changing with fO_2. As we raised in one of our earlier responses, it is possible (based on zircon geochemical data) that there could have been metasedimentary materials around for a potential Hadean impact. So having these materials present in the Sudbury target rock, and possibly indicated by our measurements of the Eu anomaly in the melt sheet zircons in not problematic.

As mentioned in our response below this one, we have also added additional text about the Eu anomalies for Sudbury zircons and for other zircon populations in the supplemental. We also show the Eu anomalies for Sudbury whole rocks. These values are 0.87 to 1.04 for mafic and felsic norite respectively while the whole rock granophyre has a stronger negative Eu/Eu* near 0.66 (a result previously reported in ref. [19]). Most of the Sudbury melt sheet zircons have $(Eu/Eu^*)_{CHUR}$ < 0.4 and the median $(Eu/Eu^*)_{CHUR}$ of the Sudbury norite zircons used for this work are 0.1 and 0.05 for the Sudbury granophyre zircons implying a reduced system, significant crystallization of plagioclase or significant melt fractionation.

We agree with the reviewer, ultimately, using zircons from both Sudbury and many other terrestrial impact craters would be ideal over single type example. However, we started with Sudbury since it is old, large, well-preserved, well-studied, and accessible. It is essentially the best type example we have for starting with. As argued throughout this response, we maintain that among known and accessible terrestrial impact structures, the Sudbury basin currently represents the most robust analogue available for a Hadean impact event. Given the scarcity of the Hadean record, we assume that any zircons derived from impact events likely originated from massive, high-energy systems capable of generating large, differentiated melt sheets analogous to the Sudbury Igneous Complex. We have included the necessary caveats throughout the manuscript, specifically clarifying that this model serves as a representative analogue for impacts into continental-like crustal targets.

L119: I wouldn't say that the difference in Eu anomalies is discussed in the supplement. It is described. I see no interpretations (and discussion implies interpretations).

We have added additional discussion (including interpretations) in the supplemental of Eu anomalies of different rock types particularly focused on the Sudbury granophyre and norite zircons. We also produced a new **Figure S13** focused on Sudbury whole rocks and the Eu anomaly. We also added **Table S4** to this section of the supplemental which shows Eu anomaly median and averages for different zircon population along with the expected zircon Eu anomaly ranges based on crystallization sequence from ref. [20]. We have also further discussed Eu anomalies in the response above this one. Here is the additional text that has been added to that section:

"Particularly, whole rock Eu for the Sudbury granophyre shows stronger Eu anomaly that for the norite layer (**Figure S13d**). While Eu/Eu* is varies from 0.87 to 1.04 for the mafic and felsic norite, the granophyre shows a much stronger negative Eu anomaly near 0.66. This result was previously reported by ref. [19] who used it, at the time, to argue for two separate parental melts for Sudbury Igneous Complex. Changes in Eu concentration and anomaly for zircons from different populations (**Figure S14**) can be driven by changes in the fO_2 but using this as an fO2 sensor is complicated by the crystallization of plagioclase depleting the melt in Eu (refs. [21,22,23])."

L127: Yet most argue that the Sudbury melt sheet started as a single parental melt. Therriault et al., 2002, Econ Geol, 1521-1540. So, what is your evidence that the parent melt was variable?

While the melt may have started out as a single melt and this is not a hypothesis we specifically disagree with, it underwent differentiation during crystallization into the different layers, which have been shown to have distinct chemistry (e.g. ref. [1]; and ref. [24] mentioned in this comment). We have sampled those different layers which show different major rock mineralogy and even different zircon trace element geochemistry in this study. Even with the evolving chemical composition of the zircon that's been recorded in these different layers, they still exhibit distinct trace element characteristics when compared to the I, S, and A type zircon trace element geochemistry. This is an important aspect of the study, which demonstrates that crystallization of an impact melt sheet into distinct layers does not mimic or emulate the trace element geochemical pattern of endogenously produced zircon.

We changed wording here and added an additional caveat in the text since we do not necessarily disagree with Sudbury starting out as single parental melt. We also feel that our machine learning results are more impactful if the single parent melt hypothesis is assumed. While there were groups who argued for separate parent melts for SIC (e.g.

ref. [19,25]) based on the Eu anomaly of the rocks of the two layers or particular field observations, ref. [24] argued for single impact melt and point to the consistent crustal isotopic signature throughout the melt sheet and differentiation sequence, among other observations.

L139: Considering the notorious problem with lots of laboratories measuring quantifiable La concentrations in zircon, can you specify why you chose a La < 1 ppm threshold?

Quantifiable amounts of La > 1 ppm generally indicate an inclusion in the zircon or significant non-zircon component to the measurement. Additionally, we wanted to be as consistent as possible in applying filtering the data to the machine learning datasets including both new data we collected and data we used from literature sources. Therefore we note that the compiled dataset of ref. [26] for I-, S-, A- zircons that we made use of for our endogenous case was also filtered by a La < 1 ppm threshold. Our other threshold LREE-I > 30 comes from ref. [27]. We added language to the manuscript itself to specify this reason.

L157: I disagree. The underlying rock types were probably quite different (as I mentioned earlier as a comment on L52).

We acknowledge that there are no preserved Hadean rocks and therefore the underlying rock types of this early period can be debated. We have made a case in our first response to the main paragraph of the review and in our response to the comment on L52, that the target rocks have similarities to TTGs and therefore the target materials that might be expected in a Hadean impact. Please see the new section added in the main text at the end of the introduction and the new **Figure 2**.

L160: Good analogues require similar boundary and starting conditions. The dominant control on trace elements in zircon in any igneous system is the parental melt. Can you justify that the parental melt compositions for the SIC and the Hadean melt sheets were similar? If not, can you at least infer that the crystallization sequences would be similar so that the imprint of various minerals you mention in your manuscript that control the investigated ratios (e.g. garnet on L271).

First, while we acknowledge the limitations brought up in the response on Line 157 and Line 52, we note that we consider Sudbury to be the best existing analogue for a Hadean impact. We have emphasized this in our responses here and in the manuscript. Since there are no preserved Hadean rocks, it is impossible to know exactly the rock types present in the Hadean. We have made the point in our response to the L52 comment that Sudbury is a possible and even likely analogue to a Hadean impact melt

sheet. Second we do think there are more similarities between the SIC and a potential impact-induced Hadean melt sheet parental compositions than there are differences. As raised throughout this response document, the sodic crystalline target rocks at Sudbury, such as the Levack gneiss and Cartier batholith, offers some analogy with a TTG-like crust. A new section has been added to the manuscript at the end of introduction that makes this case (i.e. **Figure 2**). Finally, zircons are currently our only window into the Hadean. This means that Hadean melt sheet composition can only be inferred via zircon geochemistry. Ultimately, this would require assuming that Hadean zircons are from impact melt sheets in the first place, or by modeling, which would then still need to be grounded in measurements.

L174: I don't follow the 'even then' logic in this sentence. If you train your algorithm on a different case study (like a TTG), then would the results not be different? And many studies of early Earth (Hadean and Eoarchean) argue that the upper continental crust (susceptible to an impact) was more mafic. TTGs are usually emplaced in the middle crust (and may or may not have participated). This would have implications for the U/Yb, Th/U, and Gd/Yb of the parental melt sheet, the crystallization sequence, and the resultant composition of zircon that crystallized from this magmatic system. So, is this really analogous to the Sudbury scenario? Perhaps I am missing a link here, but this sentence (which is a key link in this manuscript) seems to mislead the reader and suggest equivalence between the early Earth and the Sudbury example regardless of the fundamental geological differences in rock types.

First, while some studies do imply a more mafic Hadean upper continental crust (refs. [28,29,30]) and sometimes even no felsic crust (ref. [31]), this is not a consensus viewpoint as many other studies and the existence of Hadean zircons themselves also argue for a less mafic early continental crust and at least some, to possibly extensive, felsic components (e.g. refs. [32,33,34,35]). As mentioned in our first response of this review, the sodic, silica-rich, infracrustal nature of the Sudbury target rocks such as Levack Gneiss has geochemistry analogous to TTGs of early Earth. Meaning that our exogenous case could roughly be interpreted (i.e. as an analogue) of an impact into TTG like material.

While machine learning can always yield new insights as the size of the dataset increases, and including more classifications could be a further extension of this work, we chose what we felt best represented most endogenous melting on Earth, which we consider to be typified by I-, S-, A- zircons and used Sudbury to build a zircon impact melt dataset that what we believe best represents exogenous melting. There are already important caveats in the manuscript, such as Sudbury representing an impact into continental crust. Our updated petrologic analysis (**Figure 2**) also implies some compositional similarity between some of the target rocks at Sudbury and Archean TTGs. Thus, we consider Sudbury the best preserved impact into continental crust and

considering that wholesale melting in large impacts probably results in similar melt sheets, we feel reasonably confident in this result. As such, our results strongly indicate that the preserved early Earth zircon record does not contain zircons from impacts into continental crust like material. We have rephrased the text of the ‘even then’ sentence to avoid overstating our case and better communicate our results and their implications.

A broad interpretation of our work might be that there was limited amounts of continental crust on the early Earth. A more limited inference that corresponds only to the results of our work would be that the early Earth zircon record does not currently contain zircons from melt sheets that formed from impacts into continental crust like material.

Reviewer #2 (Remarks to the Author):

Overall this is a welcome and thorough investigation using high quality geochemical inputs and a novel machine-learning application to address a longstanding debate. The authors have extrapolated their model, trained on the trace element composition of Sudbury melt sheet zircons, to test for evidence of Hadean melt sheets (targets unknown). It is proposed that the negative outcome of their test indicates that the Jack Hills Zircon suite is not impact-derived. I found this to be a bit of an overreach. As the authors acknowledge in L168-175, their Sudbury training model does not capture zircons produced by melt sheets derived from OCEANIC crust. They are correct that their model is best suited to impact melting of continental crust; "Even then, our results indicate that the zircons analyzed are not sourced from impacts into continental crust like material.L174". So, I support publication of this work, but with a less expansive conclusion and title along the lines of "No Evidence for Continent-derived Impact Melt Sheets from the Early Earth Detrital Zircon Record". This more limited implication is not the authors' fault, as there are no easily accessible melt sheet zircons from oceanic crust targets available for training, to the best of my knowledge...the Mjolnir impact in North Sea is perhaps the best potential target but drill cores from the Norwegians are not easily accessed. In the absence of natural samples, or a modelled data set for zircon chemistries from ocean-crust-derived impact melting, the authors might consider limiting the scope of their conclusions. Otherwise it is a sound and valuable contribution that is more rigorous than most other recent examinations of Jack Hills zircon geochemistry in this light.

We acknowledge the limitations brought up by reviewer #2. To address this we accept their proposed change to the title to include ‘Continent-derived’ when referring to the early earth melt sheets. We have also specified ‘continent-derived’ in the text of the manuscript at various relevant locations. Finally, we changed language of some sentences in the discussion to avoid an overly expansive interpretation while still discussing the important implications of our research.

References cited in this Response to Reviewers document:

1. Lightfoot, P. C. *Nickel Sulfide Ores and Impact Melts: Origin of the Sudbury Igneous Complex*.
2. Grieve, R. A. F., Ames, D. E., Morgan, J. V. & Artemieva, N. The evolution of the Onaping Formation at the Sudbury impact structure. *Meteorit & Planetary Scien* **45**, 759–782 (2010).
3. Grieve, R. A. F. & Cintala, M. J. An analysis of differential impact melt-crater scaling and implications for the terrestrial impact record. *Meteoritics* **27**, 526–538 (1992).
4. Grieve, R. A. F., Stöffler, D. & Deutsch, A. The Sudbury structure: Controversial or misunderstood? *J. Geophys. Res.* **96**, 22753–22764 (1991).
5. Grimes, C. B. *et al.* Trace element chemistry of zircons from oceanic crust: A method for distinguishing detrital zircon provenance. *Geol* **35**, 643 (2007).
6. Grimes, C. B., Wooden, J. L., Cheadle, M. J. & John, B. E. “Fingerprinting” tectono-magmatic provenance using trace elements in igneous zircon. *Contrib Mineral Petrol* **170**, 46 (2015).
7. Kirkland, C. L., Smithies, R. H., Taylor, R. J. M., Evans, N. & McDonald, B. Zircon Th/U ratios in magmatic environs. *Lithos* **212–215**, 397–414 (2015).
8. Wielicki, M. M., Harrison, T. M. & Schmitt, A. K. Geochemical signatures and magmatic stability of terrestrial impact produced zircon. *Earth and Planetary Science Letters* **321–322**, 20–31 (2012).
9. Kenny, G. G., Whitehouse, M. J. & Kamber, B. S. Differentiated impact melt sheets may be a potential source of Hadean detrital zircon. *Geology* **44**, 435–438 (2016).

10. Latypov, R., Chistyakova, S., Grieve, R. & Huhma, H. Evidence for igneous differentiation in Sudbury Igneous Complex and impact-driven evolution of terrestrial planet proto-crusts. *Nat Commun* **10**, 508 (2019).

11. Barrett, T. J. *et al.* A chlorine isotope transect across Sudbury Basin (Canada) impact deposits reveals systematic isotope fractionation. *Geology* **53**, 155–160 (2025).

12. Peters, D., C. Michael, L. & F. Pattison, E. VARIABLE IMPACT DEVOLATILIZATION IN THE SUDBURY IGNEOUS COMPLEX AND ITS RELEVANCE FOR Ni--Cu--(PGE) SULPHIDE ORE FORMATION. *Geoscience Canada* **52.2**, (2025).

13. Cavosie, A. J., Valley, J. W., Wilde, S. A., & E.I.M.F. Magmatic δ18O in 4400–3900 Ma detrital zircons: A record of the alteration and recycling of crust in the Early Archean. *Earth and Planetary Science Letters* **235**, 663–681 (2005).

14. Trail, D. *et al.* Constraints on Hadean zircon protoliths from oxygen isotopes, Ti-thermometry, and rare earth elements. *Geochem Geophys Geosyst* **8**, 2006GC001449 (2007).

15. Harrison, T. M. Hadean Jack Hills Zircon Geochemistry. in *Hadean Earth* 143–178 (Springer International Publishing, Cham, 2020). doi:10.1007/978-3-030-46687-9_7.

16. Cherniak, D. J., Hanchar, J. M. & Watson, E. B. Rare-earth diffusion in zircon. *Chemical Geology* **134**, 289–301 (1997).

17. Cherniak, D. J., Hanchar, J. M. & Watson, E. B. Diffusion of tetravalent cations in zircon. *Contributions to Mineralogy and Petrology* **127**, 383–390 (1997).

18. Ariskin, A. A., Deutsch, A. & Ostermann, M. Sudbury Igneous Complex: Simulating phase equilibria and in situ differentiation for two proposed parental magmas. in

Large meteorite impacts and planetary evolution; II (Geological Society of America, 1999). doi:10.1130/0-8137-2339-6.373.

19. Chai, G. & Eckstrand, R. Rare-earth element characteristics and origin of the Sudbury Igneous Complex, Ontario, Canada. *Chemical Geology* **113**, 221–244 (1994).
20. Yakymchuk, C., Holder, R. M., Kendrick, J. & Moyen, J.-F. Europium anomalies in zircon: A signal of crustal depth? *Earth and Planetary Science Letters* **622**, 118405 (2023).
21. Trail, D., Bruce Watson, E. & Tailby, N. D. Ce and Eu anomalies in zircon as proxies for the oxidation state of magmas. *Geochimica et Cosmochimica Acta* **97**, 70–87 (2012).
22. Drake, M. J. The oxidation state of europium as an indicator of oxygen fugacity. *Geochimica et Cosmochimica Acta* **39**, 55–64 (1975).
23. Drake, M. J. & Weill, D. F. Partition of Sr, Ba, Ca, Y, Eu2+, Eu3+, and other REE between plagioclase feldspar and magmatic liquid: an experimental study. *Geochimica et Cosmochimica Acta* **39**, 689–712 (1975).
24. Therriault, A. M., Fowler, A. D. & Grieve, R. A. F. The Sudbury Igneous Complex: A Differentiated Impact Melt Sheet. *Economic Geology* **97**, 1521–1540 (2002).
25. Johns, G. W. & Dressler, B. O. The Sudbury Igneous Complex--An Impact Melt Sheet? in vol. 26 679 (1995).
26. Zhong, S. H. *et al.* A machine learning method for distinguishing detrital zircon provenance. *Contrib Mineral Petrol* **178**, 35 (2023).

27. Bell, E. A., Boehnke, P. & Harrison, T. M. Recovering the primary geochemistry of Jack Hills zircons through quantitative estimates of chemical alteration. *Geochimica et Cosmochimica Acta* **191**, 187–202 (2016).

28. Reimink, J. R. Petrogenesis of the Acasta Gneiss Complex, Northwest Territories, Canada. (University of Alberta Library, 2016). doi:10.7939/R3FB4WV19.

29. O'Neil, J. & Carlson, R. W. Building Archean cratons from Hadean mafic crust. *Science* **355**, 1199–1202 (2017).

30. O'Neil, J., Carlson, R. W., Francis, D. & Stevenson, R. K. Neodymium-142 Evidence for Hadean Mafic Crust. *Science* **321**, 1828–1831 (2008).

31. Rollinson, H. There were no large volumes of felsic continental crust in the early Earth. *Geosphere* **13**, 235–246 (2017).

32. Harrison, T. M. The Hadean Crust: Evidence from >4 Ga Zircons. *Annu. Rev. Earth Planet. Sci.* **37**, 479–505 (2009).

33. Harrison, T. M., Bell, E. A. & Boehnke, P. Hadean Zircon Petrochronology. *Reviews in Mineralogy and Geochemistry* **83**, 329–363 (2017).

34. Boehnke, P. *et al.* Potassic, high-silica Hadean crust. *Proc. Natl. Acad. Sci. U.S.A.* **115**, 6353–6356 (2018).

35. Bernadet, J. *et al.* Making continental crust on water-bearing terrestrial planets. *Sci. Adv.* **11**, eads6746 (2025).
