# Supplementary material for: A missing continental impact melt sheet signal in the early Earth detrital zircon record
Source: Nat Commun. 2026 Aug 11;17:10106. doi: 10.1038/s41467-026-76529-w (PMC13598112; doi:10.1038/s41467-026-76529-w)
Supplement: Supplementary file 3 [file 41467_2026_76529_MOESM1_ESM.docx]

**Supplementary Information:**

This is the supplementary information for Szumila et al., 2026: **A Missing Continental Impact Melt Sheet Signal in the Early Earth Detrital Zircon Record**. Here, we provide additional details on our geochemical analyses and the methodology of our machine learning approach. We also conduct support vector machine (SVM) analysis on an oceanic and continental zircon analyses compiled from the literature (**Supplementary Data 2**) to refine the discriminant diagrams often used to classify oceanic and continental zircons^1^.

**Pre-compiling and filtering of data**

The general outline of the flow of data (**Supplementary** **Figure 1**) is that in addition to the data we gather from our LA-ICP-MS analyses for this paper, we supplemented our analyses with a number of analyses from different work. To do so, we coded a number of ‘compilers’ for different datasets that use the published supplementary files from various papers and change the formats to be more similar to the GEOROC-inspired tabular format we use. Then these are all compiled into one excel file (i.e. **Supplementary Data 1**) which is then passed through multiple filters. Handling the data in this way allows us to have fine control over how the data is processed and make sure each entry is processed correctly. For example, if one publication reports chondrite normalized values and a different entry does not, we can standardize them via the compilers so that all data is reported the same way. We also used this pre-compiling of data method to include an update (Chowdhury, personal communication) to the Hadean zircon trace element REEs^2^ analyzed for that work. In other cases, we used this step to clean the data of non-numeric inputs in columns. Since the all data is standardized via this pre-compilation, there is then no issue with converting whole columns during our analysis in the jupyter notebook SudburyZirconAnalysis_JupyterNotebookCode.ipynb. Additionally, we can only use analyses for which U/Yb, Th/U, Yb, And Gd/Yb have all been analyzed for the SVM due to the requirement of having all input fields analyzed or interpolated. However by handling the data through this pipeline, we can use a somewhat broader set of analyses for the **Figure 3** panels and analyses since we can include all relevant analyses. That is, for our REE and Ti in zircon crystallizations plots, even if a worker only analyzed a REE or Ti and not U and Th, we can still include their analysis at that step but before the machine learning.

Supplementary Figure 1. Flow of data through our code.

**Additional notes on methodology for Sudbury zircons**

**Supplementary Table 1:**

Number of Sudbury zircons / data points used in the SVM and before and after filtering

**Supplementary Table 2**

Number of zircons in the endogenous zircon classification, early earth zircon classifications, and for zircons from infilling sediments at Sudbury before and after filtering.

All data used in figures and analysis for this manuscript passes through the filters described in this main text (La < 1 ppm and LREE-I > 30 ^[3]^), in addition to filtering the authors used when reporting their data originally. **Supplementary Table 2** shows the same filtering applied as in **Supplementary Table 1** for endogenous zircons, early earth zircons for predicting on, and for zircons from infilling sediments at Sudbury. The fourth column: “Values after availability of features wanted for SVM” is only relevant for classifications that were used to train or predict on. It is still shown for the zircons from infilling sediments and footwall at Sudbury for completeness. Also, while there are additional Jack Hills zircon trace element analyses in Cavoise et al., 2006; Peck et al., 2004; Maas et al., 2002; Crowley et al., 2005 in our combined **Supplementary Data 1** that have U/Yb, Th/U, Yb (ppm), and Gd/Yb analyzed, they did not simultaneously report the ^207^Pb/^206^Pb age and so could not be used in Figure 3. When these Jack Hills zircons with U/Yb, Th/U, Yb (ppm), and Gd/Yb analyzed but lacking age data are used for predictions anyway, the total Jack Hills zircon predicted on becomes n=175. Even so, still only 1 zircon analysis from that group is identified as being from the exogenous classification.

**Correlation coefficients and variance inflation factor for Sudbury and I-, S-, A- zircons**

**Supplementary Figure 2. a)** Correlation coefficient plot of the elements and ratios used in training the SVM for our Sudbury melt sheet and endogenous zircons from **Supplementary Data 1**. **b)** Variance inflation factor(VIF) plots for the different datasets explored here. The Pearson correlation coefficient measures how much two values are linearly correlated, with a value of 1 meaning the two features are perfectly linearly correlated. The VIF provides a measure of how much an individual predictor variable’s variance is increased by multicollinearity present in the model. A general rule of thumb is that a value >0.8 in the correlation coefficient matrix or a VIF > 5 indicates the presence of multicollinearity^4,5^.

Multicollinearity occurs when multiple input variables in a model have a linear relationship with each other, making it difficult to interpret the individual contributions of the variables to the model. To investigate if multicollinearity was present in the datasets, we calculated the Pearson correlation coefficients and the variance inflation factor (VIF) of the relevant datasets (**Supplementary** **Figure 2**). Multicollinearity is not necessarily a problem if the model is purely predictive, but becomes an issue when investigating feature importance in a model since different predictors may be representing the same effect. For the Sudbury and endogenous zircons, none of the correlation coefficients are >0.8 and and the VIF is less 0.5 (**Supplementary** **Figure 2**), meaning that multicollinearity will not hamper interpretations.

**Petrologic Rationale for Trace Element Selection**

The fundamental premise of our approach is that impact-generated melts do not follow the equilibrium thermodynamic pathways typical of endogenic processes. On Earth, melt generation—such as adiabatic decompression or granitic anatexis—produces regular, reproducible trace element fractionations governed by well-understood thermodynamic constraints. In such cases, the partial melt (which later crystallizes zircon) is modeled to account for a refractory mineral residue. In contrast, a large-scale impact like Sudbury produces a near-total melt of a substantial vertical crustal column.

While many trace element ratios could be incorporated into our support vector machine (SVM) algorithm, we selected U/Yb, Th/U, Yb, and Gd/Yb due to their demonstrated diagnostic value over the last two decades. We leverage these signatures, originally derived from terrestrial melting environments, to evaluate and classify geochemical signals in impact-generated systems. Increased U/Yb is associated with crustal input or enriched mantle sources^6^, while Gd/Yb measures MREE enrichment, which may indicate garnet crystallization or titanite and apatite fractionation^6^. The Th/U ratio serves as a proxy for crystallization behavior and magma evolution^7^. Element selections featuring the U/Yb ratio and Yb were utilized by prior research showing that impact melt zircons from Sudbury, Morokweng, Manicouagan, and Vredefort plot almost exclusively within continental fields^8^. By applying our framework featuring our 4 inputs, we evaluate whether massive, homogenized melts—representing a unique mixture of diverse target lithologies—produce geochemical signals distinct from endogenous I-, S-, and A-type granitoids.

As supported by impact modeling of the Sudbury structure, which reconstructs a transient cavity diameter of ~100 km^9^, the resulting melt zone extends to a depth of approximately 30 km^10^. This indicates the sheet is derived from the bulk melting of target material—including both upper and middle crystalline crust—homogenized above the liquidus. In this total-melt scenario, a traditional restite does not exist to sequester trace elements in the same manner. This process is inherently more stochastic than conventional magmatism and is not governed by the pathways of endogenic crustal production. We therefore hypothesize that the resulting zircons will record "non-traditional" signatures—specifically in U/Yb, Th/U, and Gd/Yb—reflecting these distinct formation mechanisms.

Finally, in impact-generated melt sheets, superheating (e.g. as demonstrated by the cubic zirconia identified at the Mistastin Lake impact^11^) could lead to extensive dissolution of inherited zircon from zircon-bearing target lithologies, increasing the Zr concentration of the melt. As the system cools, this elevated Zr budget may permit zircon saturation to be achieved at higher temperatures than in conventional magmatic systems. Consequently, zircon may crystallize under different thermal and mineralogical conditions, which can influence trace element partitioning and result in distinct zircon geochemical signatures.

**Tuning of hyperparameters**

To tune the machine learning hyperparameters, we applied Grid Search. In Grid Search, the hyperparameters are tuned by training multiple models with sets of different hyperparameters, often varying on a logarithmic basis. We use the Python function GridSearchCV from the scikit-learn package^12^ to train an array of multiple models with a ‘grid’ of supplied hyperparameters. An example of this technique for geologic data can be found where GridSearchCV was applied to optimize hyperparameters for a SVM to predict landslides^13^. GridSearchCV can also implement cross-validation; for this, we used the default five-fold cross-validation. This means that for each specified set of hyperparameters, the model would be trained on a different subset of the training data and its performance evaluated by its score on the left-out data. Five-fold means this was done five times. Finally, the best model (quantified by a score from GridSearchCV) is the one we used to make our plots and predict on unseen data.

Notably, a low *C* (regularization parameter) may provide a better estimator by introducing more slack into the fit which may allow a model to classify more total samples correctly by avoiding overfitting. For the support vector machines trained on Sudbury zircons, and endogenous zircons (i.e. those sourced from I-, S-, A- granitoids), we conducted a grid search on linear kernel SVM with possible *C* from 0.01, 0.1, 1, 10, 100, 1000 and SVMs with poly, rbf or sigmoid kernels, with *C* from 0.01 to 1000 and gamma from 0.001 to 1. From this, we found that a support vector machine with an RBF kernel (*C*=1000, gamma=1) had the best performance.

**Class-balanced SVM (SVM2)**

The model (SVM1) presented in the main text trains the n=328 impact melt sheet zircon as one class and uses the I-, S-, A- zircons (n=3369) to typify an endogenous class by doing a typical 80-20 train-test split. We also trained an additional SVM model (SVM2) where we balanced these classes to a 1000 analyses each. In this case, after splitting each classification 80-20, we either oversampled or undersampled, depending on the number of analyses in the class, from the training data to a total of 1000 zircon analyses per class for training. The test sets were never over- or undersampled and just used with the quantity of measurements available after the train-test split. When applied to the zircons withheld that made up the test data, this model had an accuracy of 97% on the test Sudbury zircons, and 92% on the test I-,S-,A- zircons with a *F_1_* score of 0.68.

**Detailed performance metrics:**

Here we discuss the performance metrics (**Supplementary Table 3**) for SVM1 and SVM2. We preferred SVM1 in the main text since it has a better *F_1_* score, a better P4 metric, and a lower false positive rate than SVM2. To be counted as a True Positive, the model must have correctly identified Sudbury melt sheet zircon in the test set as being from Sudbury, while a True Negative is a correctly identified I-, S-, A- zircon from the test set. False positive would be an I-, S-, A- zircon that was incorrectly identified as being a Sudbury melt sheet zircon while False Negative represents a Sudbury zircon incorrectly identified as being from the endogenous zircon classification. The lower false positive rate of only 2.1%, as well as the higher (and closer to 1) *F_1_* score and P4 metric of SVM1, meant that we favored this model as giving more accurate results.

**Supplementary** **Table 3:** Performance metrics for SVM1 and SVM2

|  | **Zircon identification with SVM1 (Kernel: rbf, C=1000, ɣ=1, no class balancing)** |  | **SVM2 (Kernel: rbf, C=1000, gamma=1, with class balancing)** |
| --- | --- | --- | --- |
| True Positive | 52 | True Positive | 64 |
| False Positive | 14 | False Positive | 57 |
| True Negative | 660 | True Negative | 617 |
| False Negative | 14 | False Negative | 2 |
| False positive rate | 2.1% | False positive rate | 8.5% |
| False discovery rate | 21% | False discovery rate | 47% |
| *F_1_* score | 0.79 | *F_1_* score | 0.68 |
| P4 metric | 0.87 | P4 metric | 0.8 |

**Applying SVM2 to pre >3.2 Ga zircons and possible interpretations for SVM2:**

The SVM2 model identified more as Sudbury or melt sheet than SVM1, the model in the main text (**Supplementary** **Table 4**, **Supplementary** **Figure 3**). Still this is only 3 individual zircons from the Jack Hills and 8 from the Barberton Greenstone Belt. With the total amount for both localities being less than 10% each and not much higher than the false positive rate (8.6%) for this model in the case of Barberton. In general, the identification of impact melt sheet zircons with this model appears relatively evenly spread out throughout time from 4.2 Ga to 3.2 Ga, with the exception of a larger peak around 3.6 Ga (**Supplementary** **Figure 3**). These samples around 3.6 Ga come from multiple Acasta gneiss rocks^14^. Most interestingly, given the proposition that some rocks from the Acasta Gneiss, particularly the 4.02 Ga Idiwhaa gneisses, represent an impact melt sheet process^15^, the class balanced model identified ~1/3 of the Acasta Gneiss zircons as the Sudbury melt classification. While this is interesting, we do not consider it likely to be a real result, given the higher false positive rate for this model and the better performance metrics on SVM1 in general. As with Acasta gneiss, this model also identified more Bushveld zircon as being Sudbury zircon than the model in the main text. Given the similarities between Bushveld and Sudbury, it is not improbable that the model could be picking up on some similar underlying chemistry, even if the origin of events (endogenous vs exogenous) is different. Given that no locality was identified as being more than 1/3rd Sudbury impact melt zircon with this model (SVM2), we also interpret this model (SVM2) as supporting evidence that none of these localities had their zircons formed mainly by shock-induced impact melt sheets as a primary source. We also used both SVM models to evaluate Bushveld zircons. While it was proposed that Bushveld had an impact origin, this is not supported by the petrologic and field evidence^16^. Since it has been previously compared with Sudbury, we evaluated these zircons via machine learning as well and also found no strong evidence for an impact origin.

**Supplementary Table 4:**

| **Zircon identification with SVM1 (Kernel: rbf, C=1000, ɣ=1, no class balancing)** | **% Sudbury (as “exogenous zircons” archetype)** | **% I-, S-, A- (as “endogenous zircons” archetype)** |
| --- | --- | --- |
| Bushveld zircon  (n=79) | 0% (n=0) | 100% (n=79) |
| **SVM2 (Kernel: rbf, C=1000, gamma=1, with class balancing)** | **% Sudbury (as “exogenous zircons” archetype)** | **% I-, S-, A- (as “endogenous zircons” archetype)** |
| Jack Hills zircon (n=97) | 3.1% (n=3) | 97% (n=94) |
| Barberton (n=87) | 9.2% (n=8) | 91% (n=79) |
| Acasta Gneiss zircon (n=92) | 23% (n=21) | 77% (n=71) |
| Bushveld zircon (n=79) | 16% (n=13) | 84% (n=66) |

**Supplementary** **Figure 3. a)** The SVM2 model trained by class balancing the I-, S-, A- and impact melt sheet zircons to 1000 analyses each. This meant oversampling the Sudbury melt sheet zircons and undersampling the I-, S-, A- zircons.

**Additional figure for Major Element Geochemistry of Target Lithologies**

**Supplementary** **Figure 4**: A different view of **figure 2** from the main text showing materials that plot farther outside the main ternary diagram. Rocks identified as TTGs can be seen to increase along the Al+(Na+K) apex and drift farther away from the A/CNK=1 line, while the felsic norites typically stay near or below this line.

**Supporting Additional Results on Sudbury Zircons**

**Imaging of zircons**

We show example CL images of zircons from a Huronian Mississagi quartzite rock, the melt sheet granophyre, and the melt sheet norite (**Supplementary** **Figure 5**). The Mississagi zircon exhibits oscillatory zoning while the two zircons from the melt sheet show only a limited pattern in CL for the granophyre to none for the norite. It is likely these differences stem from the melting conditions during the Sudbury event itself since later metamorphism or tectonic processing would have also affected the Mississagi as well. As for Hadean zircons, they exhibit variability, displaying either limited CL banding (as in the images of Sudbury melt rock zircons below in d) and f) ) or oscillatory zoning patterns^17^. Overall, it is unlikely that CL characteristics alone can identify a zircon that grew in an impact melt sheet. However, limited CL banding may suggest a degree of chemical homogeneity which is consistent with zircons that grew in a rapidly crystallizing impact melt sheet. Zircons recovered from the melt sheet layer show frequent inclusions when compared to zircons from the target rocks, along with a less well defined crystallographic faces. We also identified various inclusions in the zircons, and present EDS spectra of these phases (**Supplementary** **Figures 6-11**).

**Supplementary** **Figure 5a)** A BSE image of a zircon from the Huronian Mississagi formation, illustrates the type of zircon present in the target rock. **(b)** BSE image of a zircon that crystallized in the granophyre layer of the melt sheet. **c)** BSE image of a zircon that crystallized in the norite layer of the melt sheet, **5d, e, f)** Corresponding CL images of the zircon in a, b and c. Each image contains a pit where laser ablation analyses were conducted.

**Quartz diorite:**

**Supplementary** **Figure 6.** EDS spectrum of a quartz inclusion in a zircon from the quartz diorite sample.

**Supplementary** **Figure 7.** EDS spectrum of an alkali feldspar inclusion in a zircon from the quartz diorite sample.

**Basal norite:**

**Supplementary** **Figure 8.** EDS spectrum showing K-Spar in a zircon from a basal norite sample.

**Supplementary Figure 9.** EDS spectrum showing titanite in a zircon from the basal norite sample.

**Supplementary Figure 10.** EDS spectrum showing plagioclase in a zircon from the basal norite sample.

**Granophyre:**

**Supplementary Figure 11.** EDS spectrum showing the spectrum from one of the backscatter bright U-Th phases in a zircon from a granophyre sample.

**Additional notes on zircon geochemistry**

**Ti-in-zircon thermometer Details for Sudbury and Hadean Zircons**

**Supplementary Figure 12.** A probability density function for Ti in zircon for Hadean zircons and Sudbury melt sheet norite and granophyre. While the data for analyses in the main text passes through all filtering, this plot was produced by specifically excluding a couple of literature reported datasets, specifically supplementary refs. ^18,19,20^ and ref. ^21^ from filtering. This was done because those works either did not report La or the elements needed to calculate the LREE-I, therefore the filtering applied in the main text excludes them for consistency. Since these works were focused on other analyses and include Ti measurements, we have purposefully included them here in this supplemental figure so that we can better compare the Ti in zircon crystallization temperatures.

The crystallization temperature of Hadean zircons is generally lower than that of Ti in zircon crystallization from other types of melts and closer to that of zircons from impact melts. This has been used to infer either wet melting conditions in the Hadean^18^, that mafic melts are an unlikely source for Hadean zircons^22^ or that impact melts are a primary source^23^. We also note the larger tail of the distribution for granophyre zircon crystallization that extends to higher temperatures. The inclusion of the additional datasets for **Supplementary Figure 12** shifts the temperature median for Hadean zircons from 686 ℃ to 691 ℃ which is not large enough to affect our interpretations.

**REE plots with whole rock concentrations and Eu Anomaly Details for Sudbury, Bushveld, Early Earth, and Hadean Zircons**

**Supplementary Figure 13a)** Minimum and maximum REE concentrations in zircons from rocks part of or near the Sudbury impact structure with Sudbury whole rocks (WR) trace element concentrations also shown as an alternate version of Figure 2a in the main text. **13b)** An alternate version of Figure 2b from the main text that still shows range of possible REE concentrations for Sudbury melt sheet zircons and also includes WR average concentrations from the north range as reported in Table 3.2 from ref. ^24^. Legends are separated from graph to increase graph visibility. **13c)** The whole rock concentrations normalized to chondrite show that a stronger negative Eu anomaly can be seen for the granophyre layer. **13d)** The Sudbury whole rock concentrations normalized to Sudbury whole rock mafic norite also shows the strongly negative Eu anomaly of the granophyre whole rock.

**Supplementary Figure 14.** This plot of the Eu anomaly vs Yb shows that Sudbury impact melt sheet zircons tend towards having a strong negative Eu anomaly (ie. (Eu/Eu*)_CHUR_ < ~0.4). While zircons from the >3.2 Ga localities have a larger range from near 1 to the same as Sudbury melt sheet zircons.

Given **figure 3c**, the pattern of distribution of Eu anomaly values for the zircons is most similar between the Hadean zircons and the Bushveld zircons, which is another line of evidence indicating an endogenous origin for the Hadean zircons. The Acasta gneiss shows a nearly bimodal distribution of potential Eu anomalies in **figure 3c** while Barberton zircons lack the same bimodality. Sudbury melt sheet zircons from both the norite and granophyre layers show distributions with centroids at larger negative Eu anomalies than any of the other zircon populations analyzed. Particularly, whole rock Eu for the Sudbury granophyre shows stronger Eu anomaly than that for the norite layer (**Supplementary Figure 13d**). While Eu/Eu* is varies from 0.87 to 1.04 for the mafic and felsic norite, the granophyre shows a much stronger negative Eu anomaly near 0.66. This result was previously reported by ref. ^25^ who used it, at the time, to argue for two separate parental melts for Sudbury Igneous Complex. Changes in Eu concentration and anomaly for zircons from different populations (**Supplementary Figure 14**) can be driven by changes in the *fO_2_* but using this as an *fO_2_* sensor is complicated by the crystallization of plagioclase depleting the melt in Eu (refs. ^26,27,28^). Additionally, we report averages for our different rock types here compared with the ranges reported from ref. ^29^ for zircons from S-type granites, A-type granites, and tonalites (**Supplementary Table 5**).

**Supplementary Table 5:**

Comparison of Eu anomaly averages and medians for zircon populations. Expected ranges based on crystallization sequences are shown for zircons A-type granites, S-type granite and tonalites from ref. ^29^

| **Compiled from the database from this work** | Eu Anomaly Average | Eu Anomaly Median | **From the analysis of ref.** ^29^ | Liquidus to solidus |
| --- | --- | --- | --- | --- |
| **Zhong et al., (2023) A-type** | 0.14 | 0.10 | **A-type granite zircons** | 0.15 to ~0.10 |
| **Zhong et al., (2023) S-type** | 0.13 | 0.13 | **S-type granite zircons** | 0.15 to ~0.3 |
| **Sudbury norite zircon** | 0.12 | 0.10 | **Tonalite zircons** | 0.5 to ~0.35 |
| **Sudbury granophyre zircon** | 0.06 | 0.05 |  |  |
| **Jack Hills zircon** | 0.25 | 0.22 |  |  |
| **Barberton zircon** | 0.29 | 0.25 |  |  |
| **Acasta gneiss zircon** | 0.38 | 0.38 |  |  |
| **Bushveld zircon** | 0.26 | 0.22 |  |  |
| **Levack gneiss zircon** | 0.57 | 0.70 |  |  |
| **Mississagi zircon** | 0.57 | 0.48 |  |  |

A strongly negative Eu anomaly can be sign that the zircon is associated with metasedimentary rock^29^ but is not a guarantee since there are many other confounding variables in the source melt (e.g. plagioclase, garnet, *fO_2_*) that will affect the that will affect the final Eu anomaly of a crystallizing zircon. We speculate that the strongly negative Eu anomalies seen here for the Sudbury norite and granophyre zircons may be due to the melt sheet differentiation since melt fractionation can result in strongly negative Eu anomalies^29,^ ^30^.

**Further trace element details for Sudbury, Early Earth, and Hadean zircons**

**Supplementary Figure 15.** Trace element plots for every unique pairing of U/Yb, Th/U, Yb (ppm), and Gd/Yb for the Sudbury norite and granophyre, and then for zircons from three early earth localities (Acasta gneiss, Barberton, and Jack Hills).

While there is some overlap between Jack Hills zircons and the Sudbury zircon analyses, there are key differences too (**Supplementary Figure 15**), with Th/U, Yb concentration and Gd/Yb generally being lower for the Jack Hills and early earth zircons. Many Acasta gneiss and Barberton zircons also plot with slightly higher values for Th/U and Gd/Yb than the Jack Hills zircons.

**Applications to oceanic and continental zircons**

Given the popularity of the discriminant lines proposed to distinguish between continental and oceanic zircons^1^, we also apply machine learning here to provide a more robust definition of these boundaries. This test also demonstrates the utility of SVMs in enhancing the accuracy and reliability of zircon classification. In detail, we use support vector machines and the publicly available oceanic (n=217-336) and continental crust (n=1259-1352) zircon analyses to derive new classification lines. The n value varies depending on the subset of element concentrations or ratios that are being analyzed (**Supplementary Table 6**). The compiled zircon analysis in **Supplementary Data 2** are from refs. ^1,31,32,33^ and ref. ^34^ which in turn compiled data from refs. ^35,36,37,38^ and ref. ^39^.

**Supplementary Table 6:**

Number of oceanic and continental zircon analyses available for machine learning in **Supplementary Data 2** considering different sets of elements.

**Correlation Coefficients and Variance Inflation Factor for oceanic and continental zircons**

**Supplementary Figure 16. a)** correlation coefficient plots for the oceanic and continental zircons from **Supplementary Data 2** when using 5 inputs. **b)** Variance inflation factor plots for the oceanic and continental zircons from **Supplementary Data 2** when using 5 inputs. **c)** correlation coefficient plots for the oceanic and continental zircons from **Supplementary Data 2** when using 4 inputs. **d)** Variance inflation factor plots for the oceanic and continental zircons from **Supplementary Data 2** when using 4 inputs.

We also calculated the correlation coefficient and VIF for Oceanic and continental zircons (**Supplementary Figure 16**) as well to establish if there was multicollinearity in the datasets since this would make interpreting individual variables’ contributions to model predictions difficult. and their relationship to the model. Therefore, since we were interested in identifying the elements that provided the most predictive power in the model, we examined the multicollinearity. There is a high degree of correlation between Y and Yb in the oceanic and continental zircons in **Supplementary Data 2** (**Supplementary Figure 16b**), meaning that including both elements in the training is likely redundant. Therefore, we trained an additional SVM using just U, Yb, Hf, U/Yb, with Y removed. As a sort of counter-example, for both **Supplementary Figure 16a** and **Supplementary** **Figure 16c**, it can be seen that the U/Yb correlation coefficient is not above 0.25 for its pairing with any other variable, meaning that the information provided by this ratio is not linearly provided by U or Yb alone.

**Machine learning with oceanic and continental zircons**

With the **Supplementary Data 2** oceanic and continental zircons, we took the log_10_ of U, Yb, U/Yb, Hf, and Y values and then trained support vector machines (SVMs) on the transformed data. A SVM is a machine learning model that aims to fit a decision boundary between two datasets and maximize the margin of a line or plane separating the datasets. In two dimensions, this reduces to the equation of a line that optimally separates the two classes. In three dimensions or more, the result becomes an equation of a plane or a hyperplane. While originally designed to work with linearly separable data, they can be applied to classes that are not linearly separable by using different kernels. For this, a ‘kernel’ is used to transform the data input to a higher-dimensional space, which enables SVMs to work with datasets that are otherwise not linearly separable. We individually trained SVMs on U vs Yb, U/Yb vs Hf and U/Yb vs Y, which are then compared to the previously reported visual fits^1^ which while utilizing the same 5 variables, only considered two variables at a time. To take advantage of the multidimensionality of this problem, we trained one SVM on Y, U, Yb, Hf, U/Yb all together and present the equation of a hyperplane (a linear plane in dimensions > 3) that separates the oceanic and continental zircons with all 5 of these inputs.

For the oceanic and continental zircon and the linear discriminant diagrams for the norite and granophyre zircons, this involved conducting a grid search with only the linear kernel and *C* from 0.1, 1, 10, 100, and 1000. The *C* value that provided the best discriminant line is labeled on the relevant plots and tables. We directly used a linear kernel when trying to refit discriminant diagrams. Applying SVMs to the oceanic and continental zircons allows the building of a multidimensional discriminant diagram. The individual discriminant diagrams of U vs Yb, U/Yb vs Y, and U/Yb vs Hf can be combined into a single multidimensional discriminant with four inputs. This method offers better accuracy in identifying oceanic zircons and continental zircons as a whole than when the diagrams are used separately. We applied SVMs as a method of investigation to enhance element discriminant diagrams by making them multidimensional. We showed how the individual discriminant can be combined into one multidimensional discriminant plane. We then applied SVM in the main text as a way of distinguishing between impact melt sheet and endogenous (from I-, S-, A- granitoids) zircons and used it to analyze the Hadean zircon record.

**Applying SVM to the continental vs oceanic zircon discriminant diagrams**

Discriminant lines that identify zircons from oceanic crust and continental crust on based on three plots of U (ppm) vs Yb (ppm), U/Yb vs Hf (ppm), and U/Yb vs Y (ppm) were previously developed[^1^](https://www.zotero.org/google-docs/?Br7Dbj). These lines, because of their ease of application and utility, have become widely applied to zircon provenance studies (e.g. refs. ^34,8,40,41^). Since these trace element concentration or trace element ratio plots mentioned above have strong discriminatory power when it comes to zircon provenance for discriminating between zircons with continental and oceanic source input, there may also be valuable in applying to exogenous versus endogenous processes.

We addressed oceanic and continental zircons using machine learning techniques to determine whether we could replicate or possibly improve upon the previous results^1^. To do this, we trained three individual support vector machines on oceanic and continental crust zircons, but only included two features that would each match the previous plots^1^ (i.e. of U vs Yb, U/Yb vs Hf , and U/Yb vs Y). Since the result of the support vector machine with a linear kernel is a line, a plane, or a hyperplane, we extracted the coefficients of our three support vector machines and plotted the discriminant line. We applied log_10_ transformations to the x and y axis before fitting with the support vector machines, and thus our discriminant lines are most accurately compared on log-log plots. The previous discriminant diagrams^1^, which used visually established lines, are defined as only two points on a log-log and semi-log plots. Thus, they cannot always be plotted with a y = mx + b form, since this would result in non-constant slopes on a log-log plot. Instead, a line defined on a log-log plot takes the form of a monomial or y=bx^m^ as seen for the U vs Yb plot. Also the plot of U/Yb vs Hf was defined with a logarithmic y-axis and linear x-axis giving it a more complex form. The U/Yb vs Y also technically takes the form of a monomial, but with m=1, so it reduces to a linear form. Accuracy on the training set for all models was always greater than 95% which shows that these classes are, by and large, linearly separable.

One huge advantage of machine learning techniques is the ability to provide new insights into multi-dimensional datasets. In detail, it becomes possible to link all five features (U, Yb, and Y, U/Yb and Hf) together and produce a hyperplane separating oceanic and continental crust zircons. We plot oceanic and continental zircon trace element data with SVM lines, and the original fits^1^ done by eye (**Supplementary Figure 17**). The equations and defining points for the lines from the original literature fit^1^ and this work are in **Supplementary Table 7**. The multidimensional fits using SVMs are in **Supplementary** **Table 8**.

**Supplementary Table 7**: Oceanic and Continental SVM results, 2d results

| Elements | Defining points^1^ | Derived functions^1^ | SVM | C | Continental Test set  Accuracy | Oceanic Test set  Accuracy |
| --- | --- | --- | --- | --- | --- | --- |
| U vs Yb | (25, 1)  (20000, 10000) | y=0.012x^1.37784^ | -5.27log(Yb ppm) + 3.03log(U ppm) + 8.55 = 0 | 10 | 100% | 90% |
| U/Yb vs Hf | (5000, 0.05)  (35000, 5) | y=(0.05) 10^((x-5000)/(35000-5000) * log10(5/0.05) )^ | -7.71log(Hf ppm) + 3.82log(U/Yb) + 33.76 = 0 | 10 | 100% | 94% |
| U/Yb vs Y | (200, 0.01)  (100000, 5) | y=0.00005x | -1.79log(Y ppm) + 2.99log(U/Yb) + 8.37 = 0 | 1 | 100% | 81% |

**Supplementary Table 8**: Oceanic and Continental SVM results, multidimensional results

| Elements and SVM kernel | SVM | C | Continental Test set  Accuracy | Oceanic Test set  Accuracy |
| --- | --- | --- | --- | --- |
| Y, U, Yb, Hf, U/Yb  (Linear kernel) | 0.84log(Y ppm) + 0.54log(U ppm) -2.81log(Yb ppm) -6.25log(Hf ppm) + 3.34log(U/Yb) + 58.5 = 0 | 10 | 99% | 97% |
| U, Yb, Hf, U/Yb  (Linear kernel) | 0.52log(U ppm) -2.18log(Yb ppm) -3.55log(Hf ppm) + 2.70log(U/Yb) + 21.18 = 0 | 1 | 99% | 95% |
| U, Yb, Hf  (Linear kernel) | -4.28log(Yb ppm) + 2.75log(U ppm) -3.37log(Hf ppm) + 19.85 = 0 | 1 | 100% | 92% |
| U, Yb, Hf  (RBF Kernel) | Not applicable | 100  (ɣ=1) | 99% | 94% |

**Supplementary Figure 17a)** The U and Yb contents of oceanic vs continental zircons plotted with both the original discriminant diagrams from the literature^1^ and a fit determined by our coded support vector machine. **17b)** The same for U/Yb and Y. **17c)** The same for U/Yb and Hf. The regions inside the dashed lines should be considered regions of lower confidence of the SVM predictions. The plots are shown with the support vector machine found to be the best classifier based on the grid search for the optimal regularization parameter (C).

**Supplementary references:**

1.	Grimes, C. B. *et al.* Trace element chemistry of zircons from oceanic crust: A method for distinguishing detrital zircon provenance. *Geol* **35**, 643 (2007).

2.	Chowdhury, W., Trail, D., Miller, M. & Savage, P. Eoarchean and Hadean melts reveal arc-like trace element and isotopic signatures. *Nat Commun* **14**, 1140 (2023).

3.	Bell, E. A., Boehnke, P. & Harrison, T. M. Recovering the primary geochemistry of Jack Hills zircons through quantitative estimates of chemical alteration. *Geochimica et Cosmochimica Acta* **191**, 187–202 (2016).

4.	Shrestha, N. Detecting Multicollinearity in Regression Analysis. *AJAMS* **8**, 39–42 (2020).

5.	Kim, J. H. Multicollinearity and misleading statistical results. *Korean J Anesthesiol* **72**, 558–569 (2019).

6.	Grimes, C. B., Wooden, J. L., Cheadle, M. J. & John, B. E. “Fingerprinting” tectono-magmatic provenance using trace elements in igneous zircon. *Contrib Mineral Petrol* **170**, 46 (2015).

7.	Kirkland, C. L., Smithies, R. H., Taylor, R. J. M., Evans, N. & McDonald, B. Zircon Th/U ratios in magmatic environs. *Lithos* **212–215**, 397–414 (2015).

8.	Wielicki, M. M., Harrison, T. M. & Schmitt, A. K. Geochemical signatures and magmatic stability of terrestrial impact produced zircon. *Earth and Planetary Science Letters* **321–322**, 20–31 (2012).

9.	Grieve, R. A. F., Ames, D. E., Morgan, J. V. & Artemieva, N. The evolution of the Onaping Formation at the Sudbury impact structure. *Meteorit & Planetary Scien* **45**, 759–782 (2010).

10.	Grieve, R. A. F. & Cintala, M. J. An analysis of differential impact melt‐crater scaling and implications for the terrestrial impact record. *Meteoritics* **27**, 526–538 (1992).

11.	Timms, N. E. *et al.* Cubic zirconia in >2370 °C impact melt records Earth’s hottest crust. *Earth and Planetary Science Letters* **477**, 52–58 (2017).

12.	Pedregosa, F. *et al.* Scikit-learn: Machine Learning in Python. https://doi.org/10.48550/ARXIV.1201.0490 (2012) doi:10.48550/ARXIV.1201.0490.

13.	Lizama, E., Morales, B., Somos-Valenzuela, M., Chen, N. & Liu, M. Understanding Landslide Susceptibility in Northern Chilean Patagonia: A Basin-Scale Study Using Machine Learning and Field Data. *Remote Sensing* **14**, 907 (2022).

14.	Reimink, J. R., Davies, J. H. F. L., Bauer, A. M. & Chacko, T. A comparison between zircons from the Acasta Gneiss Complex and the Jack Hills region. *Earth and Planetary Science Letters* **531**, 115975 (2020).

15.	Johnson, T. E. *et al.* An impact melt origin for Earth’s oldest known evolved rocks. *Nature Geosci* **11**, 795–799 (2018).

16.	Buchanan, P. C. & Reimold, W. U. Studies of the Rooiberg Group, Bushveld Complex, South Africa: No evidence for an impact origin. *Earth and Planetary Science Letters* **155**, 149–165 (1998).

17.	Cameron, E. M. *et al.* Evidence for oceans pre-4300 Ma confirmed by preserved igneous compositions in Hadean zircon. *American Mineralogist* **109**, 1670–1681 (2024).

18.	Watson, E. B. & Harrison, T. M. Zircon Thermometer Reveals Minimum Melting Conditions on Earliest Earth. *Science* **308**, 841–844 (2005).

19.	Valley, J. W., Cavosie, A. J., Fu, B., Peck, W. H. & Wilde, S. A. Comment on ‘Heterogeneous Hadean Hafnium: Evidence of Continental Crust at 4.4 to 4.5 Ga’. *Science* **312**, 1139–1139 (2006).

20.	Trail, D., Mojzsis, S. J. & Harrison, T. M. Thermal events documented in Hadean zircons by ion microprobe depth profiles. *Geochimica et Cosmochimica Acta* **71**, 4044–4065 (2007).

21.	Trail, D., Watson, E. B. & Tailby, N. D. The oxidation state of Hadean magmas and implications for early Earth’s atmosphere. *Nature* **480**, 79–82 (2011).

22.	Harrison, T. M. The Hadean Crust: Evidence from >4 Ga Zircons. *Annu. Rev. Earth Planet. Sci.* **37**, 479–505 (2009).

23.	Kenny, G. G., Whitehouse, M. J. & Kamber, B. S. Differentiated impact melt sheets may be a potential source of Hadean detrital zircon. *Geology* **44**, 435–438 (2016).

24.	Lightfoot, P. C. *Nickel Sulfide Ores and Impact Melts: Origin of the Sudbury Igneous Complex*. (Elsevier, 2017). doi:10.1016/B978-0-12-804050-8.00001-8.

25.	Chai, G. & Eckstrand, R. Rare-earth element characteristics and origin of the Sudbury Igneous Complex, Ontario, Canada. *Chemical Geology* **113**, 221–244 (1994).

26.	Trail, D., Bruce Watson, E. & Tailby, N. D. Ce and Eu anomalies in zircon as proxies for the oxidation state of magmas. *Geochimica et Cosmochimica Acta* **97**, 70–87 (2012).

27.	Drake, M. J. The oxidation state of europium as an indicator of oxygen fugacity. *Geochimica et Cosmochimica Acta* **39**, 55–64 (1975).

28.	Drake, M. J. & Weill, D. F. Partition of Sr, Ba, Ca, Y, Eu2+, Eu3+, and other REE between plagioclase feldspar and magmatic liquid: an experimental study. *Geochimica et Cosmochimica Acta* **39**, 689–712 (1975).

29.	Yakymchuk, C., Holder, R. M., Kendrick, J. & Moyen, J.-F. Europium anomalies in zircon: A signal of crustal depth? *Earth and Planetary Science Letters* **622**, 118405 (2023).

30.	Sawyer, E. W. The Role of Partial Melting and Fractional Crystallization in Determining Discordant Migmatite Leucosome Compositions. *Journal of Petrology* **28**, 445–473 (1987).

31.	Grimes, C. B. *et al.* On the occurrence, trace element geochemistry, and crystallization history of zircon from in situ ocean lithosphere. *Contrib Mineral Petrol* **158**, 757–783 (2009).

32.	Ballard, J. R., Palin, J. M. & Campbell, I. H. Relative oxidation states of magmas inferred from Ce(IV)/Ce(III) in zircon: application to porphyry copper deposits of northern Chile. *Contrib Mineral Petrol* **144**, 347–364 (2002).

33.	Coogan, L. A. & Hinton, R. W. Do the trace element compositions of detrital zircons require Hadean continental crust? *Geol* **34**, 633 (2006).

34.	Carley, T. L. *et al.* Iceland is not a magmatic analog for the Hadean: Evidence from the zircon record. *Earth and Planetary Science Letters* **405**, 85–97 (2014).

35.	Claiborne, L. L. Understanding upper crustal silicic magmatic systems using the temporal, compositional, and thermal record in zircon. (Vanderbilt).

36.	Claiborne, L. L., Miller, C. F., Flanagan, D. M., Clynne, M. A. & Wooden, J. L. Zircon reveals protracted magma storage and recycling beneath Mount St. Helens. *Geology* **38**, 1011–1014 (2010).

37.	Flanagan, D. Zircon from Swift Creek stage eruptions records the assembly and evolution of an intrusive magmatic complex beneath Mount St. Helens. (Vanderbilt, 2009).

38.	Stelten, M. E. & Cooper, K. M. Constraints on the nature of the subvolcanic reservoir at South Sister volcano, Oregon from U-series dating combined with sub-crystal trace-element analysis of plagioclase and zircon. *Earth and Planetary Science Letters* **313–314**, 1–11 (2012).

39.	Barth, A. P., Wooden, J. L., Jacobson, C. E. & Economos, R. C. Detrital zircon as a proxy for tracking the magmatic arc system: The California arc example. *Geology* **41**, 223–226 (2013).

40.	Kielman, R., Whitehouse, M., Nemchin, A. & Kemp, A. A tonalitic analogue to ancient detrital zircon. *Chemical Geology* **499**, 43–57 (2018).

41.	Morag, N. *et al.* The Origin of Plagiogranites: Coupled SIMS O Isotope Ratios, U–Pb Dating and Trace Element Composition of Zircon from the Troodos Ophiolite, Cyprus. *Journal of Petrology* **61**, egaa057 (2020).
